# Dual-Stimuli Responsive Cystamine-Modified Polydopamine Coatings as Payload Gatekeepers

**DOI:** 10.3390/molecules31142413

**Published:** 2026-07-09

**Authors:** Sylwia Ostrowska, Monika Szukowska, Yeonho Kim, Radosław Mrówczyński

**Affiliations:** 1 Faculty of Chemistry, Adam Mickiewicz University, Uniwersytetu Poznańskiego St. 8, 61-614 Poznań, Poland; 2 Department of Chemistry, Gachon University, Seongnam 13120, Republic of Korea

**Keywords:** drug carriers, polydopamine, mesoporous silica, drug release, cystamine, cross-linking

## Abstract

We present cystamine-modified polydopamine (PDA) coatings as tunable gatekeepers for mesoporous silica nanoparticles (MSNs) in drug delivery. Unlike conventional post-functionalization strategies, cystamine moieties were incorporated directly into the PDA network, enabling tunable shell composition and redox responsiveness by simply adjusting the dopamine-to-cystamine ratio. By varying the cystamine:dopamine ratio, pH- and redox-responsive release of doxorubicin (DOX) and sorafenib (SO) was achieved, with release kinetics following the Higuchi model. Cystamine-modified PDA nanoparticles with varying disulfide bridge content were synthesized and comprehensively characterized using SEM, TGA, FTIR, and zeta potential measurements. The cystamine content was found to influence thermal stability, coating performance, and protective properties. Importantly, increasing disulfide content did not necessarily improve release performance, suggesting that excessive crosslinking may partially restrict shell permeabilization and drug diffusion. These findings reveal important structure–property relationships in catechol-based coatings and underline the significance of disulfide linkages in the design of bioinspired stimuli-responsive drug delivery systems.

## 1. Introduction

According to data from the World Health Organization, cancer remains one of the leading causes of death worldwide, accounting for more than 10 million deaths in 2020 alone [1]. Conventional chemotherapy commonly employs hydrophilic drugs such as doxorubicin hydrochloride (DOX) and hydrophobic therapeutics such as sorafenib (SO) [2,3]. However, the lack of selective delivery to tumour tissues causes severe systemic toxicity and damage to healthy cells [4,5]. In addition, poor solubility and limited bioavailability of many anticancer drugs reduce therapeutic efficiency. Therefore, the development of multifunctional materials capable of efficient drug encapsulation and controlled release remains highly desirable [6,7,8]. Nanotechnology offers promising opportunities for designing advanced drug delivery systems that can improve drug solubility, enhance therapeutic efficacy, and reduce side effects through targeted delivery [9,10,11]. Various nanomaterials, including magnetic nanoparticles [12], carbon nanotubes [13], liposomes [14], gold nanoparticles [15,16], dendrimers [17,18], and carbosilicate or polyether systems [19], have been explored as drug carriers. Among them, mesoporous silica nanoparticles (MSNs) are particularly attractive because of their high surface area, tunable pore structure, thermal stability, and biocompatibility [20]. These features enable efficient loading of anticancer drugs within the mesoporous framework. To prevent premature drug leakage, MSN surfaces are frequently modified with organic shells that function as protective gatekeepers and may additionally provide stimuli-responsive release behaviour [21,22,23,24,25,26].

Importantly, tumour tissues and intracellular cancer compartments are characterized by acidic pH values, elevated glutathione (GSH) concentrations, and increased levels of reactive oxygen species (ROS) compared with healthy tissues [27,28,29]. These conditions provide attractive triggers for the development of pH- and redox-responsive drug delivery systems.

Polydopamine (PDA), a mussel-inspired biomimetic polymer, has attracted considerable attention in materials science, nanotechnology, and biomedicine because of its facile synthesis, strong adhesive properties, antioxidant activity, biodegradability, and high photothermal conversion efficiency [30,31,32]. PDA has been widely applied in photocatalysis [33], organocatalysis [34], water purification [35], antibacterial materials [36], biosensors [37], molecular imprinting [38], tissue engineering [39], and bioimaging [40]. Importantly, PDA can form stable coatings on a broad range of organic and inorganic substrates, including silica, metals, carbon nanomaterials, and polymers [30,41,42,43]. The abundance of catechol, quinone, and amine groups enables multiple interactions with molecules and metal ions through Michael addition, Schiff base reactions, and hydrogen bonding [44,45]. Furthermore, PDA coatings exhibit excellent biocompatibility and can be readily functionalized with polymers, biomolecules, and inorganic nanostructures [46,47,48,49,50,51,52,53,54,55,56,57,58].

Although PDA itself exhibits pH- and redox-responsive behaviour, its responsiveness is difficult to systematically tune. Such modifications are commonly achieved by grafting compounds including folic acid [52,53,54], poly(ethylene glycol) (PEG) [55], hyaluronic acid (HA) [56], and cysteine [57] onto pre-formed PDA coatings. Moreover, oxidation of catechol moieties to quinone groups enables the deposition of metal nanoparticles such as Au, Ag, and Pt onto PDA surfaces [58]. Incorporation of cystamine introduces additional disulfide-containing moieties that enable modulation of coating composition, degradation behaviour, and release characteristics through simple adjustment of the dopamine-to-cystamine ratio. Recently, co-polymerization of PDA with cystamine has been explored for TiO_2_ immobilization [59]. A similar strategy was employed by Wen and co-workers to fabricate smart fabrics with pH-responsive wettability through grafting of PDA and cystamine dihydrochloride [60]. In addition, Jin et al. developed cystamine-functionalized glass nanopores for selective glutathione sensing based on the Michael addition reaction between cystamine amino groups and PDA adhered to the nanopore surface [61]. Nevertheless, the preparation of cystamine-containing PDA shells for mesoporous nanocarriers that enable controlled, stimuli-responsive drug release remains largely unexplored.

In the present work, we report the synthesis of cystamine-modified polydopamine (PDA_Cyst) coatings containing built-in disulfide bridges and their application as dual-stimuli-responsive gatekeepers for mesoporous silica nanoparticles. Unlike conventional surface grafting approaches, cystamine moieties were incorporated directly into the PDA polymer network, enabling tunable shell composition and responsiveness. The developed coatings provide controlled release of both hydrophilic DOX and hydrophobic SO under tumour-relevant acidic and redox-active conditions. Simultaneously, the PDA framework ensures strong adhesion, efficient drug retention, and structural stability of the nanocarrier. Overall, the developed PDA_Cyst system represents a versatile platform for the design of pH- and redox-responsive drug delivery systems with tunable release properties for nanomedicine applications. Recent studies have highlighted the growing interest in dual-stimuli-responsive mesoporous silica- and polydopamine-based drug delivery systems capable of responding to multiple tumor-associated stimuli, including pH, redox, oxidative, thermal, and enzymatic triggers. Most reported approaches rely on post-synthetic functionalization of preformed PDA coatings or on the attachment of responsive moieties to the particle surface. Although these strategies provide stimulus responsiveness, they offer limited control over the distribution of redox-active functionalities within the PDA framework [62,63]. For example, disulfide-containing and cystamine-based PDA nanocarriers have been developed to achieve redox-responsive drug release and improved therapeutic performance. However, most reported systems rely on post-synthetic functionalization or on incorporating cystamine-containing components into complex hybrid architectures rather than directly integrating cystamine into the PDA network itself [64].

Furthermore, cystamine-modified polydopamine-coated mesoporous silica nanoparticles are developed as stimuli-responsive carriers for doxorubicin and sorafenib delivery. The influence of cystamine incorporation on the physicochemical properties and release behavior of the systems was investigated under different physiological conditions. In contrast to previous reports, the approach presented herein is based on the direct co-polymerization of dopamine and cystamine, enabling the incorporation of disulfide moieties throughout the polymer network and allowing systematic tuning of coating composition and degradation behavior. This strategy provides a simple route to dual-responsive, PDA-based coatings with tunable physicochemical properties and drug-release characteristics.

## 2. Results and Discussion

### 2.1. Synthesis and Characterization of PDA_Cyst

**Figure 1:**
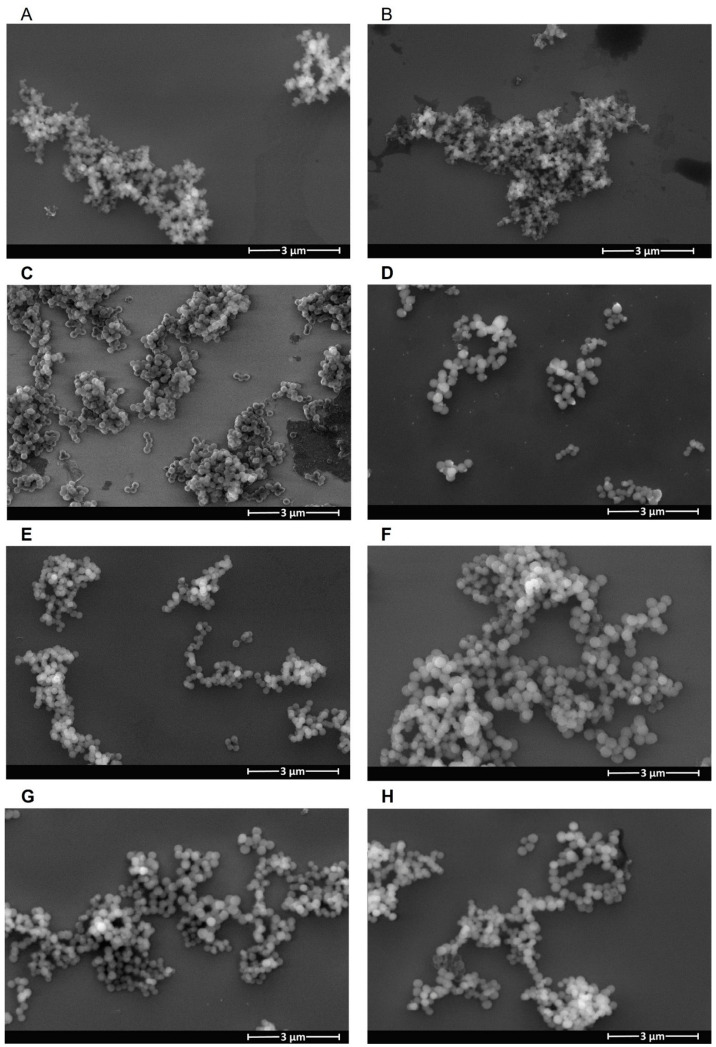
SEM images of PDA_Cyst 3:1 nanoparticles (**A**); PDA_Cyst 1:3 nanoparticles (**B**); MSN@DOX (**C**); MSN@SO (**D**); MSN@DOX@PDA_Cyst 3:1 (**E**); MSN@DOX@PDA_Cyst 1:3 (**F**); MSN@SO@PDA_Cyst 3:1 (**G**) and MSN@SO@PDA_Cyst 1:3 (**H**).

**Figure 2:**
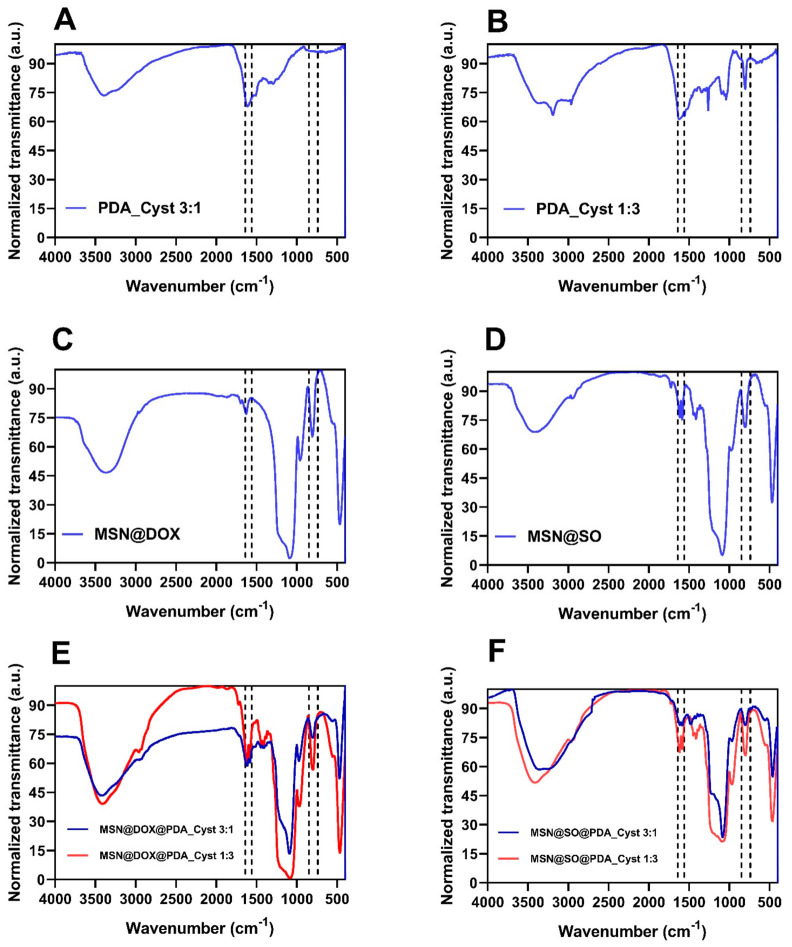
FT-IR spectrum PDA_Cyst 3:1 (**A**); PDA_Cyst 1:3 (**B**); MSN@DOX (**C**); MSN@SO (**D**); sup erimposed MSN@DOX@PDA_Cyst 3:1 and MSN@DOX@PDA_Cyst 1:3 (**E**); MSN@SO@PDA_Cyst 3:1 and MSN@SO@PDA_Cyst 1:3 (**F**).

So far, polydopamine modifications with cystamine have primarily relied on immobilizing cystamine onto the surface of pre-synthesized polydopamine coatings [59,60,61]. In the present work, we aimed to extend the scope of such modifications by directly synthesizing cystamine-modified polydopamine (PDA_Cyst) coatings in which cystamine moieties are incorporated not only at the surface but also within the internal structure of the forming polymer network. Such an approach enables the fabrication of coatings with tunable composition and redox-responsive properties resulting from the presence of built-in disulfide linkages. The development of stimuli-responsive drug carriers, particularly redox-responsive systems, is strongly justified by the biochemical environment of tumour cells. Cancer tissues are characterized by elevated levels of reactive oxygen species (ROS) and glutathione (GSH) compared with healthy tissues [65,66,67,68]. GSH, the major intracellular non-enzymatic antioxidant, plays a crucial role in maintaining cellular redox homeostasis. While extracellular GSH concentrations are relatively low (2–20 µM), intracellular levels typically range from 0.5 to 10 mM, especially in the cytosol and mitochondria [69]. These differences make redox-active groups, including disulfide linkages, particularly attractive for the design of stimuli-responsive mesoporous silica nanoparticles (MSNs) [70,71]. Initially, the feasibility of synthesizing PDA_Cyst through oxidative polymerization of dopamine hydrochloride and cystamine in Tris buffer (pH 8.5) was investigated. The first experiments were performed using dopamine:cystamine weight ratios of 3:1 and 1:3. SEM imaging revealed that both systems formed spherical nanoparticles with average diameters of approximately 240 nm and 220 nm, respectively (Figure 1A,B). These observations prompted further investigation of the influence of dopamine-to-cystamine ratio on nanoparticle morphology and size. Therefore, additional PDA_Cyst materials were synthesized using a broad range of weight ratios, including 100:1, 10:1, 5:1, 1:5, 1:10, and 1:100. SEM analysis (Figure S1A–F, Supporting Information) demonstrated that nanoparticle size strongly depended on the relative content of dopamine and cystamine within the polymer structure. Dopamine-rich systems generally produced larger nanoparticles, whereas cystamine-rich systems generated smaller particles. This trend was particularly evident for the extreme compositions, where PDA_Cyst 100:1 produced nanoparticles with an average diameter of approximately 310 nm, while PDA_Cyst 1:100 yielded particles averaging approximately 180 nm. Interestingly, although cystamine-rich compositions produced smaller nanoparticles, they also exhibited a significantly higher tendency toward aggregation. This observation was supported by dynamic light scattering (DLS) measurements, which revealed hydrodynamic diameters of approximately 350 nm for PDA_Cyst 100:1 and 540 nm for PDA_Cyst 1:100. These findings are particularly important in the context of coating mesoporous silica nanoparticles, where aggregation behaviour may strongly influence coating homogeneity, shell permeability, and cargo retention properties. Based on the obtained results, PDA_Cyst 3:1 and PDA_Cyst 1:3 were selected as representative systems for further physicochemical characterization. The FTIR spectra of these materials are shown in Figure 2A,B. In the case of PDA_Cyst 3:1, the spectrum exhibited characteristic PDA bands, including signals in the 1500–1650 cm^−1^ region assigned to N–H vibrations and a broad band at 3200–3600 cm^−1^ corresponding to hydroxyl groups. In contrast, PDA_Cyst 1:3 exhibited several additional sulfur-related bands associated with cystamine incorporation into the polymer structure. A signal observed at approximately 600 cm^−1^ was assigned to S–S stretching vibrations, while bands around 800 cm^−1^ and 1050–1013 cm^−1^ were attributed to S–OCH and S=O functionalities, respectively. Furthermore, a band at 1272 cm^−1^ was tentatively assigned to sulfur-containing functionalities. Similar to PDA_Cyst 3:1, signals corresponding to N–H and hydroxyl groups were also observed in the ranges of 1500–1650 cm^−1^ and 3186–2948 cm^−1^, respectively. Interestingly, sulfur-related absorptions were not clearly visible in the FTIR spectrum of PDA_Cyst 3:1 despite the presence of cystamine during polymerization. Therefore, elemental analysis was additionally performed to verify the incorporation of cystamine moieties into the polymer network. The obtained results (Table 1) confirmed the presence of sulfur in both investigated materials. PDA_Cyst 3:1 contained 0.66% sulfur, whereas PDA_Cyst 1:3 exhibited a substantially higher sulfur content of 4.66%. We further performed the XPS analysis of PDA_Cyst 1:3, which proved the presence of an S-S bond in the obtained materials. Importantly, the spectra of S pointed to possible oxidation of S to sulphones. The carbon spectrum deconvolution revealed the presence of C-C, C=O, and C-S as well as C=N and C-N bonds. Detailed spectra are presented in Figure S2 in Supporting Information.

Based on the results obtained from various techniques (FTIR, EA, and XPS), we were convinced that cystamine was incorporated during PDA formation. However, we were unable to unequivocally distinguish between covalent incorporation of cystamine throughout the PDA network and bound cystamine to the surface of the materials.

The zeta potential of the obtained materials was measured. The performed analysis revealed that the zeta potential values gradually increased with increasing cystamine content. At higher cystamine percentages, the zeta potential reached values of up to +6 mV, whereas strongly negative values were observed for materials with PDA-to-cystamine ratios ranging from 100:1 to 3:1 (Figure S3, Supporting Information) To further correlate the composition of PDA_Cyst coatings with their thermal properties and indirectly evaluate the degree of cystamine incorporation within the polymer network, thermogravimetric analysis (TGA) combined with derivative thermogravimetry (DTG) was performed for PDA_Cyst 3:1 and PDA_Cyst 1:3 (Figure S4A,B, Supporting Information). Both materials exhibited multistep mass-loss profiles characteristic of hybrid inorganic–organic systems. The initial weight loss observed below approximately 120–150 °C corresponded mainly to the removal of physically adsorbed water and residual solvents. The second degradation stage, occurring between approximately 200 and 350 °C, was associated with decomposition of the PDA_Cyst organic framework, including degradation of both the polydopamine backbone and cystamine-derived fragments. This process was reflected by pronounced maxima in the DTG curves corresponding to the highest mass-loss rates. At temperatures above approximately 350 °C, further decomposition and carbonization of residual organic components occurred, ultimately leading to the formation of a thermally stable inorganic residue originating from the nanoparticle core. Importantly, PDA_Cyst 1:3 exhibited a noticeably higher total mass loss than PDA_Cyst 3:1 over the entire investigated temperature range, confirming the higher organic content resulting from increased cystamine incorporation. Moreover, differences in the position and intensity of DTG maxima suggest variations in thermal stability and crosslinking density of the polymer coatings arising from the applied dopamine-to-cystamine ratio. Overall, the TGA/DTG results further confirm the successful formation of PDA_Cyst coatings and demonstrate that the composition of the polymer network can be effectively tuned by modulating the dopamine-to-cystamine ratio.

### 2.2. Synthesis and Characterization of MSN@Drug@PDA_Cyst

**Figure 3:**
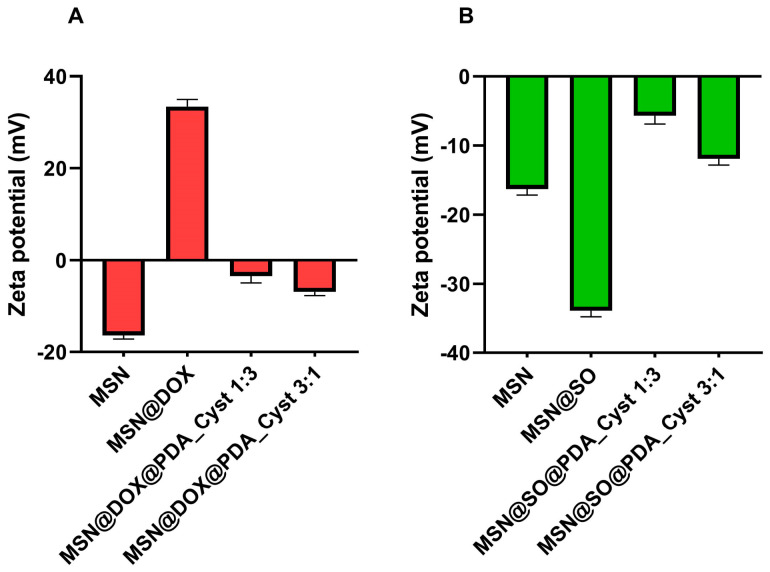
Change in zeta potential: MSN; MSN@DOX; MSN@DOX@PDA_Cyst 1:3; MSN@DOX@PDA_Cyst 3:1 (**A**); MSN; MSN@SO; MSN@SO@PDA_Cyst 1:3; MSN@SO@PDA_Cyst 3:1 (**B**).

Following the successful synthesis of cystamine-modified polydopamine polymers, these materials were further employed as stimuli-responsive gatekeeper coatings for mesoporous silica nanoparticles (MSNs). In the first stage, MSNs were synthesized using our previously reported CTAB-templated sol–gel method [72]. Calcination of the obtained materials generated the mesoporous structure. The synthesized nanoparticles exhibited a negative surface charge with a zeta potential value of approximately −15 mV (Figure 3). SEM analysis of bare MSNs (Figure S5A, Supporting Information) revealed spherical nanoparticles with an average diameter of approximately 200 nm. The corresponding FTIR spectra of pristine MSN and PDA, presented in Figure S5 of the Supporting Information, were consistent with previously reported data. Hydrophilic doxorubicin hydrochloride (DOX) and hydrophobic sorafenib (SO) were subsequently loaded into the mesoporous structure using a diffusion-driven loading approach [72,73,74]. The drug-loaded nanoparticles were then dispersed in dopamine hydrochloride and cystamine dihydrochloride solutions under mildly alkaline conditions, resulting in direct in situ polymerization of PDA_Cyst shells on the MSN surface. By varying the dopamine-to-cystamine ratio during polymerization, a series of MSN@DRUG@PDA_Cyst systems with tunable shell composition, permeability, and stimuli-responsive release properties was obtained.

### 2.3. MSN@DOX@PDA_Cyst

In our experiments, hydrophilic doxorubicin hydrochloride (DOX) was selected as a model anticancer drug because of its widespread clinical use in the treatment of various cancers, including hepatocellular carcinoma and breast cancer [2,3]. DOX was loaded into the mesoporous structure of MSNs in PBS buffer at pH 7.4. Neutral conditions were chosen to maintain a balance between DOX stability and solubility while simultaneously promoting efficient diffusion of the drug into the mesoporous channels. The loading capacity of DOX, determined by UV–Vis spectroscopy, reached approximately 66 wt.%.

To confirm successful drug loading and subsequent formation of the PDA_Cyst coating, FTIR analysis was performed for MSN@DOX and MSN@DOX@PDA_Cyst systems. The FTIR spectra of MSN@DOX (Figure 2C), MSN@DOX@PDA_Cyst 3:1 (Figure 2E), and MSN@DOX@PDA_Cyst 1:3 (Figure 2E) exhibited characteristic bands at approximately 800 cm^−1^ and 1100 cm^−1^ assigned to Si–O–Si vibrations, while the signal around 960 cm^−1^ originated from silanol groups (Si–OH) present on the silica surface. Signals corresponding to DOX were also observed, including bands associated with C–O/C–O–C vibrations and characteristic aromatic and carbonyl functionalities. A broad absorption band in the 3400–3500 cm^−1^ range was attributed to hydroxyl groups originating from both MSNs and DOX molecules.

Following coating with PDA_Cyst, additional absorption bands appeared at approximately 1600 cm^−1^ and 1571 cm^−1^, corresponding to aromatic C=C vibrations and N–H bending modes characteristic of the polydopamine framework. These changes confirm successful formation of the PDA_Cyst shell on the MSN surface. Although sulfur-related bands originating from cystamine moieties were not clearly distinguishable in the MSN@DOX@PDA_Cyst spectra, most likely because of overlap with intense silica absorptions, the results obtained in the previous section, including FTIR and elemental analysis of PDA_Cyst nanoparticles, strongly support successful incorporation of cystamine-containing coatings onto the MSN surface.

Additional TEM analyses of MSN@PDA_Cyst 1:3 and MSN@PDA_Cyst 3:1 (Figures S7 and S9, Supporting Information) revealed the formation of polydopamine-based coatings on the MSN particles. Furthermore, the corresponding EDX elemental mapping (Figures S8 and S10, Supporting Information) confirmed the presence and homogeneous distribution of carbon, nitrogen, oxygen, and sulfur within the analyzed structures. These results support the incorporation of cystamine-derived moieties into the PDA_Cyst material, in agreement with the structural characterization of the corresponding non-MSN PDA_Cyst materials.

Additional confirmation of both DOX loading and PDA_Cyst coating formation was provided by zeta potential measurements (Figure 3A). Bare MSNs exhibited a zeta potential of −15.7 ± 0.6 mV, which changed to +32.3 ± 0.8 mV after DOX loading, confirming successful incorporation of positively charged DOX molecules into the mesoporous structure. Subsequent coating with PDA_Cyst resulted in a decrease in the zeta potential to −6.8 ± 0.2 mV for MSN@DOX@PDA_Cyst 3:1 and −2.4 ± 0.4 mV for MSN@DOX@PDA_Cyst 1:3. For comparison, the previously reported MSN@DOX@PDA system exhibited a zeta potential of −18.6 ± 0.4 mV [69], indicating that incorporation of cystamine into the PDA framework significantly influences the surface properties of the resulting coating. SEM analysis further demonstrated that neither DOX loading nor PDA_Cyst coating formation significantly affects particle morphology or induces structural deformation. The nanoparticles retained their spherical morphology and comparable particle size after coating (Figure 1C–F).

Moreover, the successive modification steps were accompanied by distinct colour changes in the materials. Bare MSNs appeared white, DOX-loaded particles exhibited a brick-red colour, whereas PDA_Cyst-coated systems became dark brown to black, providing additional visual confirmation of successful coating formation.

### 2.4. MSN@SO@PDA_Cyst

Sorafenib (SO) was selected as a model hydrophobic anticancer drug approved in the US and Europe for the treatment of advanced renal cell carcinoma, liver cancer, and radioactive iodine-resistant advanced thyroid carcinoma [75]. Due to the insolubility of SO in water, we used DMF to load Sorafenib into MSN, which can be easily removed by water extraction (details are provided in the Section 3). The loading capacity determined by UV-Vis analysis of SO was 65.2 wt.%. In contrast to DOX, the loading of SO molecules did not change the colour of MSNs. However, coating MSNs@SO with PDA_Cyst nanoparticles yielded black particles. The MSN@SO and MSN@SO@PDA_Cyst FTIR spectra are shown in Figure 2D,F. FTIR spectra of MSN@SO show bands at ~800 cm^−1^ and ~1100 cm^−1^ which are assigned to Si-O-Si vibrations. The vibration band at about 960 cm^−1^ originates from silanol groups (Si-OH). The stretching bands observed at 1602 cm^−1^, 1721 cm^−1^ (C=O stretch), and 3061 cm^−1^ were assigned to functional groups from SO, such as carbonyl groups, amines, and amides. This confirms successful loading of SO into the mesoporous silica nanoparticles. After the PDA_Cyst coating, new absorption signals at 1522 cm^−1^ and 1465 cm^−1^ appeared in the spectrum of MSN@SO@PDA and were assigned to the C=C resonance vibrations in the aromatic ring and the NH bending vibrations from PDA moiety. A broad band of about ~3400–3500 cm^−1^ came from the -OH group in Si-OH catechol moieties. In this case, also in the FTIR spectra, it was difficult to assign bands that would characterize the cystamine group present in the polymer coating, which is caused by their obscuring by intense bands from MSN.

Successful SO loading and subsequent PDA_Cyst shell formation were further corroborated by changes in the zeta potential values. The zeta potential shifted from −15.7 ± 0.6 mV for bare MSNs to −33.2 ± 0.7 mV after SO loading and subsequently increased to −12.1 ± 0.2 mV for MSN@SO@PDA_Cyst 3:1 and −6.1 ± 0.2 mV for MSN@SO@PDA_Cyst 1:3 (Figure 3B). It should be noted that the zeta potential for the MSN@SO@PDA carrier where the polydopamine coating was not modified in any way was −15.2 ± 0.2 mV [69]. Similarly, the previously reported MSN@SO@PDA system was used as a reference material for evaluating the influence of cystamine incorporation on the release behavior of sorafenib-loaded carriers [69]. The SEM image of MSN@SO, MSN@SO@PDA_Cyst (3:1) and MSN@SO@PDA_Cyst (1:3) shows that the particles retained the spherical structure and their size was also not influenced by PDA_Cyst coating (Figure 1D,G,H).

### 2.5. Drug Release from MSN@Drug@PDA_Cyst Under Different Physiological Conditions

#### 2.5.1. DOX Release from MSN@DOX@PDA_Cyst

**Figure 4:**
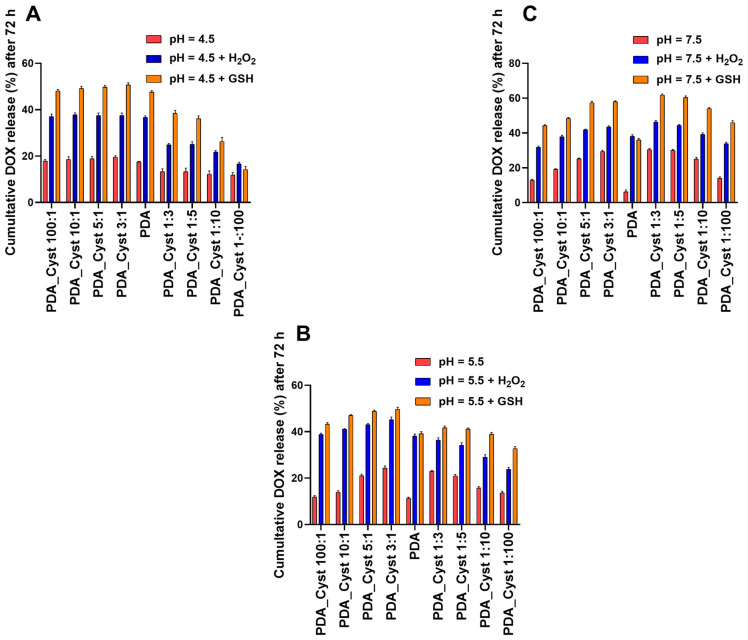
Comparison of DOX release from MSN@DOX@PDA_Cyst carriers prepared using different dopamine:cystamine feed ratios under different release conditions; 4.5; pH 4.5 + 10 mM H_2_O_2_; pH 4.5 + 10 mM GSH (**A**); pH 5.5; pH 5.5 + 10 mM H_2_O_2_; pH 5.5 + 10 mM GSH (**B**); pH 7.5; pH 7.5 + 10 mM H_2_O_2_; pH 7.5 + 10 mM GSH (**C**).

When designing anticancer drug delivery systems, a crucial aspect is the evaluation of drug release profiles under conditions that mimic the tumour microenvironment. Therefore, release studies were performed in buffer solutions with different pH values (4.5, 5.5, and 7.5), corresponding to lysosomal, endosomal, and physiological conditions, respectively. In addition, oxidative and reductive stress conditions were simulated by supplementing the buffers with hydrogen peroxide (H_2_O_2_) and glutathione (GSH), respectively. Elevated concentrations of GSH are characteristic of intracellular tumor environments, whereas 10 mM H_2_O_2_ was used here as an accelerated oxidative stress model to evaluate the responsiveness of the PDA_Cyst coating under strongly oxidizing conditions [59,61,76]. A concentration of 10 mM GSH was selected to mimic the elevated intracellular glutathione levels commonly reported for cancer cells. First, the pH-triggered release of DOX from MSN@DOX@PDA_Cyst was investigated at pH 4.5, 5.5, and 7.5 using a stirring procedure in glass flasks at 37 °C. The amount of released drug in the supernatant was quantified by UV–Vis spectroscopy using the standard calibration curve method [69]. The cumulative release profiles of DOX after 72 h for all investigated variants are presented in Figure 4. The influence of cystamine incorporation was evaluated by comparison with previously reported MSN@DOX@PDA carriers coated with unmodified polydopamine, which served as the reference system throughout this study [69]. The loading and release properties of the PDA-coated carriers were investigated under comparable experimental conditions in our previous work, enabling direct assessment of the effect of cystamine incorporation into the PDA network. It should be noted that the investigated formulations represent two different composition ranges. In the PDA_Cyst 100:1–3:1 series, the dopamine content was gradually decreased while the amount of cystamine remained constant. In contrast, in the PDA_Cyst 1:100–1:3 series, the cystamine content was gradually decreased while the dopamine amount remained constant. Therefore, the observed release behaviour reflects changes in the relative dopamine-to-cystamine ratio rather than the effect of cystamine content alone.

Analysis of the obtained data revealed a strong dependence of drug release efficiency on the composition of the MSN@DOX@PDA_Cyst carriers prepared with different PDA:Cyst feed ratios. For MSN@DOX@PDA_Cyst carriers prepared with dopamine:cystamine feed ratios of 100:1, 10:1, 5:1, and 3:1, the highest amount of released DOX was consistently observed for the PDA_Cyst 3:1 coating, regardless of the release environment. Moreover, under most release conditions, particularly at pH 5.5 and 7.5 as well as under redox-responsive conditions, an increase in DOX release was observed with increasing cystamine incorporation into the PDA_Cyst coating (PDA_Cyst 100:1 < 10:1 < 5:1 < 3:1). However, this trend was less pronounced under strongly acidic conditions (pH 4.5), indicating that the effect of cystamine incorporation depends on the release environment. Importantly, comparison with MSN@DOX@PDA coated with unmodified PDA demonstrated that increasing cystamine incorporation enhanced the release efficiency [75]. Even the MSN@DOX@PDA_Cyst 100:1 sample, containing only a small amount of cystamine, exhibited improved release compared with the pure PDA-coated carrier. This effect was particularly pronounced under physiological and redox-responsive conditions, namely at pH 7.5, pH 7.5 + H_2_O_2_, and pH 7.5 + GSH (Figure 4). A similar trend was observed for carriers prepared with a higher cystamine content, labelled as PDA_Cyst 1:100, 1:10, 1:5, and 1:3. Among these materials, the PDA_Cyst 1:3 coating exhibited the highest cumulative DOX release under all tested conditions. In this case, the release efficiency followed the order: PDA_Cyst 1:100 < 1:10 < 1:5 < 1:3. Compared with the unmodified PDA-coated carrier [69], lower DOX release values were observed for these samples under acidic conditions (pH 4.5, pH 4.5 + H_2_O_2_, and pH 4.5 + GSH), whereas comparable release profiles were obtained at pH 5.5. In contrast, a substantial increase in DOX release was observed at pH 7.5 and in the presence of H_2_O_2_ or GSH, indicating enhanced responsiveness of the PDA_Cyst coating under physiological and redox-active environments. Notably, the relative contribution of dopamine and cystamine within the coating strongly influenced the release behavior depending on the environmental conditions. Under acidic conditions, carriers containing a higher proportion of dopamine groups released larger amounts of DOX than carriers enriched with cystamine groups. However, this trend was reversed at pH 7.5 and under redox-responsive conditions (pH 7.5 + H_2_O_2_ and pH 7.5 + GSH). This behavior is particularly evident when comparing corresponding coating compositions such as PDA_Cyst 3:1 versus 1:3 or PDA_Cyst 5:1 versus 1:5. These findings clearly demonstrate that the structure and composition of the PDA_Cyst coating play a critical role in regulating drug retention and release, thereby providing a versatile platform for tuning the release behavior of silica-based drug delivery systems (Figure 4). The release behavior of the developed PDA_Cyst coatings depends on both pH and the composition of the coating. Therefore, the incorporation of cystamine influences the permeability and responsiveness of the PDA_Cyst coating, resulting in release profiles that differ from those typically observed for conventional pH-responsive systems.

**Figure 5:**
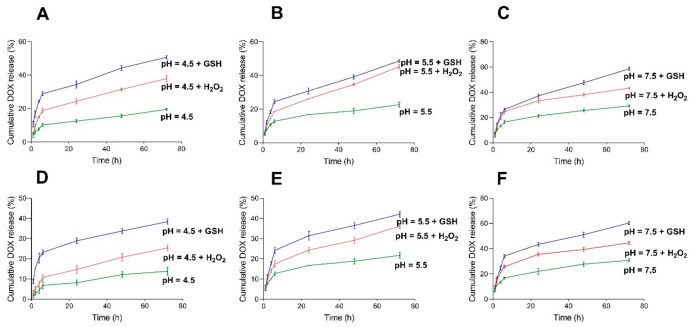
DOX release kinetic curves from MSN@DOX@PDA_Cyst 3:1 (**A**–**C**) and DOX release kinetic curves from MSN@DOX@PDA_Cyst 1:3 (**D**–**F**).

We further analysed drug release kinetics in detail using MSN@DOX@PDA_Cyst 3:1 and MSN@DOX@PDA_Cyst 1:3 as representative systems, as these carriers exhibited the highest cumulative DOX release. Moreover, these formulations enabled a direct comparison of dopamine- and cystamine-dominated coatings and their influence on release behavior. Analysis of the release kinetics for MSN@DOX@PDA_Cyst 3:1 (Figure 5A–C), in which dopamine-derived groups dominate the coating structure, revealed that increasing pH promoted DOX release. The cumulative release values after 72 h reached 19.2% in citrate buffer at pH 4.5, 23.7% at pH 5.5, and 28.71% in Tris buffer at pH 7.5. The addition of 10 mM H_2_O_2_ further enhanced the release efficiency, increasing the cumulative release to 36.64% in citrate buffer at pH 4.5, 44.33% at pH 5.5, and 42.98% in Tris buffer at pH 7.5. A similarly pronounced effect was observed in the presence of glutathione (GSH), which is elevated in cancer cells and plays a key role in maintaining redox homeostasis. After the addition of 10 mM GSH, the cumulative DOX release values increased to 49.96% in citrate buffer at pH 4.5, 49.10% at pH 5.5, and 57.69% in Tris buffer at pH 7.5. These results clearly demonstrate the strong redox responsiveness of the PDA_Cyst 3:1 coating. Comparison of these results with previously reported MSN@DOX@PDA carriers [69] further highlights the advantages of cystamine-modified coatings. It should also be noted that unmodified PDA exhibits intrinsic responsiveness toward oxidative and reductive environments due to the presence of catechol and quinone groups. Consequently, increased drug release is also observed for PDA-coated carriers in the presence of H_2_O_2_ and GSH. The incorporation of cystamine does not introduce redox responsiveness itself but rather enhances and modulates the response through the presence of disulfide linkages. For MSN@DOX@PDA, the cumulative release values were 17.4% at pH 4.5, 10.9% at pH 5.5, and 5.5% at pH 7.5. In the presence of H_2_O_2_, the release increased to 46.0%, 37.1%, and 27.2% for pH 4.5, 5.5, and 7.5, respectively, whereas in the presence of GSH the corresponding values were 47.1%, 38.5%, and 35.4%. These findings demonstrate that incorporating cystamine into the PDA structure provides an effective strategy for tuning drug-release properties and modulating the coating response under different pH and redox conditions. A direct comparison with MSN@DOX@PDA_Cyst 1:3 (Figure 5D–F), where cystamine-derived groups dominate the shell structure, revealed distinct release behavior. Under acidic conditions, lower cumulative release values were observed compared with MSN@DOX@PDA_Cyst 3:1, reaching 12.51% at pH 4.5 and 22.78% at pH 5.5. In contrast, in Tris buffer at pH 7.5, the cumulative DOX release increased to 29.97%, slightly exceeding the value obtained for MSN@DOX@PDA_Cyst 3:1. A similar trend was observed under oxidative conditions. For MSN@DOX@PDA_Cyst 1:3, the cumulative DOX release values in the presence of 10 mM H_2_O_2_ were 24.45% at pH 4.5, 35.33% at pH 5.5, and 45.50% at pH 7.5. In GSH-containing media, the release values further increased to 37.48%, 41.11%, and 61.29%, respectively. Notably, the highest cumulative release among all investigated systems was observed for MSN@DOX@PDA_Cyst 1:3 in Tris buffer containing 10 mM GSH. The obtained results demonstrate the crucial role of the PDA_Cyst coating in regulating drug retention and release from mesoporous silica carriers. The incorporation of cystamine introduces disulfide bonds into the PDA network, providing additional redox-responsive characteristics and significantly altering the degradation behavior of the coating. In dopamine-rich coatings such as PDA_Cyst 3:1, partial degradation of the PDA framework under acidic conditions likely facilitates drug diffusion and release [56,76]. In contrast, coatings enriched with cystamine groups exhibit enhanced responsiveness under neutral and redox-active conditions. We hypothesize that the increased release observed for PDA_Cyst 1:3 in Tris buffer at pH 7.5 is associated with partial cleavage or destabilization of disulfide bonds in the presence of hydroxyl ions and elevated temperature. Such processes may promote coating degradation and increase shell permeability, thereby facilitating DOX diffusion from the carrier. Consequently, the higher release observed for PDA_Cyst 1:3 compared with PDA_Cyst 3:1 or unmodified PDA under physiological and redox-responsive conditions can be attributed to the increased susceptibility of the cystamine-rich coating to structural rearrangement and degradation.

In addition to their pH-responsive behaviour, the developed cystamine-modified polydopamine carriers also exhibited pronounced sensitivity toward redox-active environments. This was confirmed by the enhanced drug release observed in the presence of H_2_O_2_ or GSH, respectively. Elevated concentrations of both compounds are characteristic features of tumour cells and are closely associated with oxidative stress and altered intracellular redox homeostasis [58,77,78,79,80].

The introduction of H_2_O_2_ into the release buffers significantly enhanced drug release from MSN@drug@PDA_Cyst carriers under all investigated pH conditions. This effect is likely associated with oxidation of catechol and phenolic groups within the PDA framework to quinone-containing structures, which may weaken intermolecular interactions between DOX and the PDA shell, including π–π stacking and hydrogen bonding interactions. As a consequence, increased permeability of the polymer coating and facilitated drug diffusion can occur.

Moreover, H_2_O_2_ may additionally interact with disulfide-containing fragments present within the PDA_Cyst shell, promoting oxidation of S–S-containing moieties to partially oxidized sulfur species, which may contribute to loosening of the polymer network structure [40]. Importantly, under near-neutral and mildly alkaline conditions (pH 7.5), H_2_O_2_ may exert an additional effect on PDA_Cyst shell destabilization. Reactions between RS–SR groups and H_2_O_2_ in the presence of OH^−^ ions may promote partial disulfide cleavage and formation of oxidized sulfur species, thereby facilitating shell permeabilization and drug leakage (see Figure 4 and Figure 5). The designed silica carriers coated with PDA_Cyst also responded efficiently to glutathione. In the performed experiments, approximately 30% higher DOX release was observed in GSH-containing buffer solutions compared with the corresponding environments without GSH (see Figure 4 and Figure 5). The redox responsiveness of PDA_Cyst-coated carriers is directly related to the dual nature of the coating structure. Disulfide bonds embedded within the polymer network may undergo reduction in the presence of GSH, leading to cleavage of S–S linkages and formation of thiol-containing species. This process likely promotes gradual swelling and partial degradation of the polymer shell, thereby enabling more efficient drug diffusion and release (Figure 4 and Figure 5). The ability of glutathione to induce cleavage of disulfide-containing structures has previously been described for nucleophilic coatings based on N-arachidonoyl dopamine (NADOPAMe) [81] as well as cystine-modified polydopamine systems [82]. Furthermore, the enhanced release observed in the presence of GSH may also be associated with increased drug solubility combined with progressive degradation and swelling of the PDA_Cyst coating, which can lead to the formation of defects and cracks within the polymer layer, facilitating drug escape from the carrier surface [83,84,85,86]. It should be noted that the enhanced drug release observed in the presence of GSH and H_2_O_2_ provides indirect evidence of the responsiveness of the PDA_Cyst coating. However, the present study does not directly demonstrate cleavage or oxidative disruption of the disulfide-containing network. Therefore, the proposed mechanism is based on the observed release behavior together with literature reports describing the redox sensitivity of disulfide-containing materials. It is worth emphasizing that, in most investigated environments, DOX release from MSN@DOX@PDA_Cyst carriers in the presence of 10 mM H_2_O_2_ was slightly lower than that observed in GSH-containing media. The corresponding data are summarized in Figure 4 and Figure 5. Nevertheless, the obtained results clearly demonstrate that DOX release from PDA_Cyst-coated MSN carriers is strongly dependent on both pH and redox conditions and can be significantly enhanced by the presence of either GSH or hydrogen peroxide.

The introduced disulfide bonds are expected to respond to elevated GSH levels and oxidative stress, increasing shell permeability and facilitating drug release. To further investigate the influence of redox-active stimuli on the PDA_Cyst material, additional TEM and SEM analyses were performed after 72 h incubation under selected release conditions. TEM images of MSN@PDA_Cyst 1:3 and MSN@PDA_Cyst 3:1 incubated in pH 4.5 buffer containing GSH (Figures S11 and S12, Supporting Information) together with SEM images obtained after incubation in pH 4.5/GSH, pH 4.5/H_2_O_2_, and pH 7.5/GSH media (Figures S13 and S14, Supporting Information) revealed morphological changes in the PDA_Cyst structures, supporting their responsiveness toward reductive and oxidative environments.

Importantly, the obtained results also indicate that higher ratio of cysteamine in the coating structure does not necessarily improve drug release efficiency. Highly crosslinked PDA_Cyst networks may require greater stimuli to induce shell loosening and permeabilization, thereby partially limiting drug diffusion from the mesoporous carrier (see Figure 4).

To determine the DOX release kinetics, the obtained data were fitted to various kinetic models, including zero-order, first-order, Higuchi’s model, Korsmeyer-Peppas model, and Hixson-Crowell model. The correlation coefficient was calculated to define the approximation accuracy of each model and the results are shown in Table 2.

The highest values of R^2^ were noted for Higuchi model in the case of MSN@DOX@PDA_Cyst 3:1 when the drug release studies were performed at pH 4.5 + 10 mM H_2_O_2_. However, under other conditions, pH 4.5, 5.5, and 7.5; pH 4.5, 5.5, and 7.5 with the addition of GSH; and pH 4.5 and 5.5 with the addition of H_2_O_2_, the R^2^ value was slightly lower. However, in all investigated environments, the R^2^ value close to one corresponded to the Higuchi release model. As for the previous DOX system, the highest R^2^ values for MSN@DOX@PDA_Cyst 1:3 release at different pH 4.5, 5.5, and 7.5 and with additions of GSH and H_2_O_2_ corresponded to the Higuchi model. Higuchi model as mentioned earlier describes the release of drugs from an insoluble matrix as a square root of a time-dependent process based on Fickian diffusion. Moreover, the Higuchi model indicates that our delivery system exhibits the highest concentration of the released drug at the initial stage of the process, and then the equilibrium state is established [87].

Comparison of the kinetic models describing DOX release from MSN@DOX@PDA_Cyst carriers with those previously reported for MSN@DOX@PDA systems containing unmodified polydopamine shells revealed that both systems follow a Higuchi-type release mechanism [69]. These findings indicate that incorporation of cystamine moieties into the polydopamine network significantly modifies shell responsiveness and permeability while preserving the dominant diffusion-controlled release behaviour.

#### 2.5.2. SO Release from MSN@SO@PDA_Cyst

**Figure 6:**
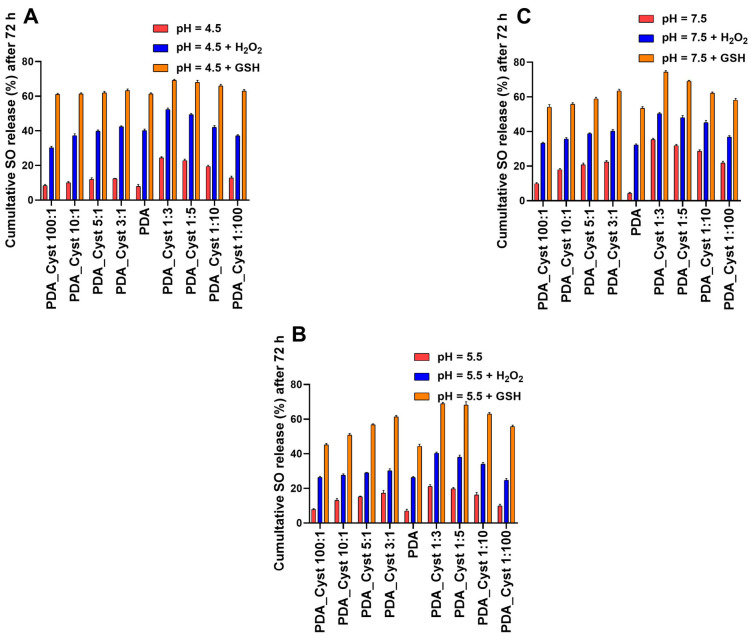
Comparison of SO release from MSN@DOX@PDA_Cyst carriers prepared using different dopamine:cystamine feed ratios under different release conditions; pH 4.5; pH 4.5 + 10 mM H_2_O_2_; pH 4.5 + 10 mM GSH (**A**); pH 5.5; pH 5.5 + 10 mM H_2_O_2_; pH 5.5 + 10 mM GSH (**B**); pH 7.5; pH 7.5 + 10 mM H_2_O_2_; pH 7.5 + 10 mM GSH (**C**).

Following the investigation of DOX release, the release profile of hydrophobic sorafenib (SO) from MSN@SO@PDA_Cyst carriers was evaluated under analogous experimental conditions. Drug release studies were performed at pH 4.5, 5.5, and 7.5 at 37 °C. Additionally, environments containing H_2_O_2_ and GSH were employed to simulate oxidative and reductive conditions characteristic of the tumour microenvironment. The amount of released SO was quantified by UV–Vis spectroscopy using the standard calibration curve method [69]. The cumulative release profiles of SO after 72 h under different physiological conditions are presented in Figure 6. The influence of cystamine incorporation was evaluated by comparison with the previously reported MSN@SO@PDA carrier coated with unmodified polydopamine, which served as the reference system throughout this study [69]. Analysis of the obtained results revealed a clear relationship between the composition of the PDA_Cyst coating and the release efficiency of SO. In the case of dopamine-rich systems, including PDA_Cyst 100:1, 10:1, 5:1, and 3:1, the highest release values were consistently observed for MSN@SO@PDA_Cyst 3:1 regardless of the investigated release environment, including acidic, physiological, and redox-responsive conditions in the presence of H_2_O_2_ or GSH. Moreover, a gradual increase in SO release was observed with increasing cystamine incorporation according to the following trend: 100:1 < 10:1 < 5:1 < 3:1. A similar tendency was observed for cystamine-rich systems (PDA_Cyst 1:100, 1:10, 1:5, and 1:3), where MSN@SO@PDA_Cyst 1:3 exhibited the highest cumulative SO release values. In this case, the following relationship was observed: 1:100 < 1:10 < 1:5 < 1:3.

**Figure 7:**
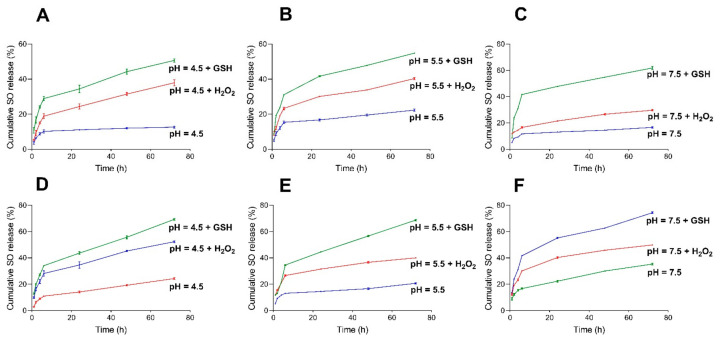
SO release kinetic curves from MSN@SO@PDA_Cyst 3:1 (**A**–**C**) and SO release kinetic curves from MSN@SO@PDA_Cyst 1:3 (**D**–**F**).

Comparison with the previously reported MSN@SO@PDA system [69] demonstrated that even a relatively low amount of cystamine resulted in a noticeable increase in SO release compared with unmodified PDA coatings. This effect was particularly pronounced under physiological and redox-responsive environments, including pH 7.5, pH 7.5 + H_2_O_2_, and pH 7.5 + GSH. Notably, a more detailed analysis of the release kinetics presented in Figure 7 revealed that the relative content of dopamine and cystamine influenced not only the cumulative amount of released drug but also the release behaviour itself. Interestingly, direct comparison of representative formulations revealed a distinct trend under acidic conditions. This effect is particularly evident when comparing MSN@SO@PDA_Cyst 1:3 and MSN@SO@PDA_Cyst 3:1, for which SO release values at pH 4.5 reached 23.84% and 12.20%, respectively. The same behaviour was also maintained under oxidative and reductive conditions, with the most pronounced differences appearing in GSH-containing media. In the case of MSN@SO@PDA_Cyst 1:3, the highest SO release value among all investigated systems was obtained (73.84% at pH 7.5 + GSH).

These results suggest that a higher content of disulfide bridges increases the susceptibility of the PDA_Cyst shell to structural changes occurring under reductive environments, resulting in enhanced shell permeability and facilitated drug release. Interestingly, the observed release behaviour partially differs from that previously observed for DOX. This finding suggests that the hydrophobic nature of SO and its interactions with the PDA_Cyst shell, including hydrophobic and π–π interactions, may influence drug retention and subsequent release in a different manner. Overall, the obtained results clearly demonstrate that modulation of the dopamine-to-cystamine ratio represents an effective strategy for regulating shell permeability and controlling the release of hydrophobic drugs from mesoporous silica carriers.

Although the cumulative release studies clearly showed the influence of coating composition and environmental conditions on SO release efficiency, they did not provide direct insight into the underlying release mechanism. Therefore, to better understand the transport behaviour of SO through the PDA_Cyst shell, the experimental data were further analysed using commonly applied kinetic models, including zero-order, first-order, Higuchi, Korsmeyer–Peppas, and Hixson–Crowell models. The fitting accuracy was evaluated using correlation coefficient (R^2^) values, and the obtained results are summarized in Table 3. For the MSN@SO@PDA_Cyst 3:1 system, the best fit was observed for the Higuchi model, particularly under pH 4.5 conditions in the presence of 10 mM H_2_O_2_. Although slightly lower fitting parameters were obtained for other investigated environments, including pH 4.5, 5.5, and 7.5 with or without the addition of GSH or H_2_O_2_, the Higuchi model consistently provided the highest correlation coefficients among all tested models. A similar tendency was observed for the MSN@SO@PDA_Cyst 1:3 carrier, where the Higuchi model also exhibited the best agreement with the experimental data under all investigated release conditions. The Higuchi model describes drug release from insoluble matrices as a diffusion-controlled process following square-root-of-time kinetics based on Fickian diffusion [88]. Therefore, the obtained results indicate that SO release from PDA_Cyst-coated MSN carriers is primarily governed by diffusion through the polymer shell, particularly during the initial stages of the release process before gradual equilibration of the system occurs. Importantly, comparison with the previously reported MSN@SO@PDA carrier containing a pure PDA shell [69] revealed that incorporation of cystamine groups into the PDA framework significantly alters shell responsiveness and permeability without changing the dominant diffusion-controlled release mechanism. These findings demonstrate that modulation of the dopamine-to-cystamine ratio enables effective tuning of release efficiency and environmental sensitivity while preserving controlled SO transport from the mesoporous silica carrier system.

## 3. Experimental

### 3.1. Materials and Characterization Methods

Tetraethyl orthosilicate (TEOS) (99.0%), sodium hydroxide (NaOH), Hexadecyltrimethylammonium bromide (CTAB) (95%), ammonium hydroxide solution (28.0–30.0%), ethyl alcohol anhydrous (99.9%)**,** ethylene glycol (EG), and L-Glutathione reduced were purchased from Sigma-Aldrich (St. Louis, MO, USA). Doxorubicin hydrochloride (DOX, 98%) was supplied by Apollo Scientific Company (Bredbury, UK). Sorafenib (SO) was supplied by Angene Chemical Company (London, UK). Cystamine dihydrochloride was provided by Fluorochem (Hadfield, UK). Dopamine·HCl was provided by Fluorochem (Hadfield, UK). Trizma base, phosphate-buffered saline tablet supplied by Sima-Aldrich Poland (Poznań, Poland). Citric acid, sodium citrate, dimethylformamide, and hydrogen peroxide solution (H_2_O_2_) were purchased from POCH S.A. (Avantor Performance Materials Poland, Gliwice, Poland). All solvents (GR grade) were used without further purification.

Elemental analysis (CHNS) was performed using a FLASH 2000 elemental analyzer (Thermo Fisher Scientific, Waltham, MA, USA) based on the dynamic combustion technique. UV–Vis absorption spectra were recorded using an Evolution 201 UV–Vis spectrophotometer ((Thermo Fisher Scientific, Waltham, MA, USA). Differential scanning calorimetry (DSC) measurements were carried out using a DSC 8500 (PerkinElmer, Waltham, MA, USA). Thermogravimetric analysis (TGA) was performed using a TGA 4000 ((PerkinElmer, Waltham, MA, USA). Dynamic light scattering (DLS) and zeta potential measurements were carried out using a Zetasizer Nano ZS (Malvern Panalytical, Malvern, UK). DLS measurements were performed using Milli-Q water as the dispersing medium. Ultrapure water (18.2 MΩ·cm at 25 °C) was obtained from a Milli-Q Direct-Q 3 UV water purification system (Millipore SAS, Molsheim, France) and was used in all experiments.Scanning electron microscopy (SEM) and energy-dispersive X-ray spectroscopy (EDS) analyses were performed using an FEI Quanta 250 FEG scanning electron microscope (FEI, Hillsboro, OR, USA). High-resolution transmission electron microscopy (HRTEM) was carried out using a JEOL ARM 200F microscope (JEOL Ltd., Tokyo, Japan). X-ray photoelectron spectroscopy (XPS) measurements were performed using a SPECS multi-chamber ultra-high vacuum (UHV) analytical system (SPECS Surface Nano Analysis GmbH, Berlin, Germany). All centrifugation steps were performed using a Frontier™ FC5515R refrigerated centrifuge ((OHAUS Corporation, Parsippany, NJ, USA) equipped with a fixed-angle rotor (24 × 1.5/2.0 mL).

### 3.2. Synthesis of Cystamine-Modified Polydopamine Nanoparticles (PDA_Cyst)

Dopamine hydrochloride (90 mg) and cystamine dihydrochloride (Cyst, 30 mg) were placed in a 250 mL round-bottom flask and dissolved in 120 mL of Tris buffer (10 mM, pH 8.5). The mixture was allowed to polymerize at room temperature (25 °C) for 16 h. The resulting PDA_Cyst (3:1) nanoparticles were washed three times with Tris buffer (pH 8.5) and Milli-Q water, then centrifuged at 16,100× *g* for 20 min at 10 °C, and finally stored in water for further use. Nanoparticles with different PDA:Cyst ratios were synthesized using the following feed compositions: PDA_Cyst (100:1), PDA_Cyst (10:1), PDA_Cyst (5:1), PDA_Cyst (1:1), PDA_Cyst (1:5), PDA_Cyst (1:10), PDA_Cyst (1:3), and PDA_Cyst (1:100). The PDA_Cyst designations refer to the feed mass ratios of dopamine hydrochloride to cystamine dihydrochloride used during synthesis and do not represent the experimentally determined composition of the resulting polymer. In each case, an appropriate amount of buffer was used to maintain a final concentration of 1 mg/mL.

### 3.3. Synthesis of Mesoporous Silica Nanoparticles (MSNs)

MSNs were synthesized by a modified Stöber method, according to previously reported procedures with slight modifications [88,89]. Briefly, hexadecyltrimethylammonium bromide (CTAB, 3 mM) was dissolved in 600 mL of deionized (DI) water and stirred for 30 min at 40 °C. Subsequently, 250 mL of ethanol and 4.7 mL of an ammonium hydroxide solution were added to the above solution, and the mixture was stirred for 5 min. Tetraethyl orthosilicate (TEOS, 4.7 mL) was then added dropwise, and the mixture was stirred for 48 h at 60 °C. After cooling to room temperature, the product was collected by filtration through a 0.22 μm membrane filter and washed with ethanol. The resulting solid MSNs were dried in an oven at 60 °C overnight. To remove CTAB, 2 g of the as-synthesized MSNs were placed in a tube furnace and calcined at 600 °C for 4 h in air.

#### 3.3.1. Loading DOX on MSNs

Doxorubicin (DOX, 10.4 mg) was dissolved in 10 mL of phosphate-buffered saline (PBS, 10 mM, pH 7.4) at room temperature (RT) using ultrasound treatment for 5 min. Subsequently, MSNs (5 mg) were ultrasonically dispersed into the solution, and the mixture was stirred magnetically at 25 °C for 24 h. The resulting MSN@DOX particles were collected by centrifugation and gently washed with Milli-Q water until no detectable DOX signal was observed in the supernatant. Finally, the product was resuspended in water and stored at 4 °C for up to 1 day before use. The amount of DOX loaded onto the MSN@DOX was quantified by UV–Vis spectroscopy at 486 nm using a standard calibration curve.

#### 3.3.2. Loading SO on MSNs

Sorafenib (SO, 10.4 mg) was dissolved in 10 mL of dimethylformamide (DMF) at room temperature (RT) using ultrasound treatment for 5 min. Subsequently, MSNs (5 mg) were ultrasonically dispersed in the solution, and the mixture was stirred magnetically at 25 °C for 24 h. The resulting MSN@SO particles were collected by centrifugation and gently washed with Milli-Q water until no detectable SO signal was observed in the supernatant. Finally, the product was resuspended in water and stored at 4 °C for up to 1 day before use. The amount of SO loaded onto MSN@SO was quantified by UV–Vis spectrophotometry at 264 nm using a standard calibration curve. A minimal amount of DMF was required to improve the solubility of SO in aqueous medium and to enable reliable UV–Vis measurements. The supernatant was initially turbid but became clearer after the addition of DMF.

The drug loading capacity was calculated using the following formula:

$$loading\;capacity\;\left(wt.\%\right)=\frac{mass\;of\;total\;drug-mass\;of\;drug\;in\;supernatant}{mass\;of\;total\;drug\;carrier}\times 100\%$$

### 3.4. Preparation of Cystamine-Modified Polydopamine Coated MSNs@drug (DOX or SO) Particles

MSN@drug (15 mg) was ultrasonically dispersed in 20 mL of Tris buffer (10 mM, pH 8.5) for 10 min. Dopamine hydrochloride (1–100 mg) and cystamine dihydrochloride (Cyst, 1–100 mg) were then added to the dispersion, and the suspension was stirred continuously at 25 °C for 18 h. Cystamine-modified polydopamine (PDA_Cyst) layers were obtained using different PDA_Cyst layers were obtained using different PDA:Cyst ratios (100:1, 10:1, 5:1, 3:1, 1:3, 1:5, 1:10, and 1:100). The notation MSN@drug@PDA_Cyst X:Y refers to MSN-based carriers coated with PDA_Cyst synthesized using the corresponding dopamine:cystamine feed ratio (X:Y). The resulting MSN@drug@PDA_Cyst nanoparticles were collected by centrifugation, washed once with Tris buffer (pH 8.5, 10 mL) and three times with Milli-Q water (10 mL each). Finally, the products were resuspended in water and stored at 4 °C for up to 1 day before use.

### 3.5. Preparation of Polydopamine Coated MSNs@drug (DOX or SO) Particles

The synthesis of MSN@drug@PDA was performed according to a previously described procedure [72]. In brief, MSN@drug (DOX or SO, 15 mg) was ultrasonically dispersed in 20 mL of Tris buffer (10 mM, pH 8.5) for 10 min. Dopamine hydrochloride (10 mg) was then added to the dispersion, and the suspension was stirred continuously at 25 °C for 18 h. The resulting MSN@drug@PDA nanoparticles were collected by centrifugation, washed once with Tris buffer (pH 8.5, 10 mL) and three times with Milli-Q water (10 mL each). Finally, the products were resuspended in water and stored at 4 °C for up to 1 day before use.

### 3.6. Drug Release from MSN@drug@PDA_Cyst Carriers Under Different Physiological Conditions

Briefly, MSN@drug@PDA_Cyst nanoparticles (DOX or SO, 2 mg) were dispersed in 2 mL of the appropriate buffer under different conditions: citrate buffer (10 mM, pH 4.5); citrate buffer (10 mM, pH 4.5) with 10 mM GSH; citrate buffer (10 mM, pH 4.5) with 10 mM H_2_O_2_; citrate buffer (10 mM, pH 5.5); citrate buffer (10 mM, pH 5.5) with 10 mM GSH; citrate buffer (10 mM, pH 5.5) with 10 mM H_2_O_2_; Tris buffer (10 mM, pH 7.5); Tris buffer (10 mM, pH 7.5) with 10 mM GSH; and Tris buffer (10 mM, pH 7.5) with 10 mM H_2_O_2_. The samples were incubated at 37 °C to investigate the effects of pH and redox agents on drug release.

Aliquots were collected at predetermined time intervals (1, 2, 4, 6, 24, 48, and 72 h), and the withdrawn volume was replaced with fresh buffer to maintain sink conditions. To evaluate the influence of mixing, experiments were conducted both in 5 mL flasks with magnetic stirring in an oil bath at 37 °C and in 2 mL tubes without stirring.

The concentration of released DOX and SO in the supernatants was quantified by UV–Vis spectroscopy at 486 nm and 264 nm, respectively. Release experiments were performed in triplicate for each condition. The results are expressed as mean ± standard deviation (SD). All release experiments were performed in triplicate, and the results are presented as mean ± SD. To gain insight into the release behavior, the experimental data were fitted using several commonly applied kinetic models. Among the tested models, the Higuchi equation generally provided the best fit to the experimental data. However, considering the stimuli-responsive nature of the PDA_Cyst coating, the release process is likely influenced not only by diffusion but also by degradation, erosion, and possible swelling of the coating. Therefore, the Higuchi model is used here primarily as a useful empirical tool for comparing release profiles rather than as evidence of an exclusively diffusion-controlled release mechanism. To analyze the drug release kinetics and mechanism, the experimental data were fitted to five mathematical models: zero-order (% drug release vs. time), first-order (log % drug remaining vs. time), Higuchi model (% drug release vs. square root of time), Korsmeyer–Peppas model (log % drug release vs. log time), and Hixson–Crowell model (cube root of % drug remaining vs. time) [73,90]. The correlation coefficient (R^2^) was calculated for each model.

## 4. Conclusions

In summary, cystamine-modified polydopamine (PDA_Cyst) coatings containing built-in disulfide linkages were developed as dual-stimuli-responsive gatekeepers for mesoporous silica nanoparticles. Unlike conventional surface functionalization approaches, cystamine moieties were directly incorporated into the PDA network, enabling tunable shell composition and responsiveness by simply adjusting the dopamine-to-cystamine ratio. Release studies using hydrophilic doxorubicin hydrochloride (DOX) and hydrophobic sorafenib (SO) demonstrated efficient, environment-dependent control of cargo release under tumour-relevant acidic, oxidative, and reductive conditions. The highest release efficiencies for both drugs were observed under reductive conditions in the presence of glutathione, confirming the pronounced redox responsiveness of the PDA_Cyst coatings. Importantly, the release profiles of both drugs were best described by the Higuchi model, indicating that diffusion through the polymer shell represents the dominant mechanism governing cargo transport. Interestingly, the obtained results revealed that increasing the content of disulfide linkages does not necessarily improve release performance, as excessive crosslinking within the polymer network may partially restrict shell permeabilization and drug diffusion. These findings demonstrate that precise modulation of PDA_Cyst composition represents an effective strategy for designing tunable stimuli-responsive coatings with controllable release properties. Overall, the developed PDA_Cyst system represents a versatile platform for designing advanced mesoporous silica-based drug delivery systems and highlights the potential of cystamine-modified polydopamine materials for future nanomedicine applications. Future work will include biological evaluation of the developed nanocarriers, including cellular uptake and cytotoxicity studies, in order to assess their biological performance, safety profile, and therapeutic applicability.

## Figures and Tables

**Table 1:** Elemental analysis (determinations of the content of C; H; N; S) for the nanoparticles.

Material	N %	C %	H %	S %
PDA_Cyst 3:1	8.74	53.37	4.11	0.66
PDA_Cyst 1:3	9.17	54.12	3.86	4.66

**Table 2:** Kinetic models used to describe the release of Doxorubicin (DOX) from materials obtained.

Receptor Medium	Zero-Order	First-Order	Higuchi	Hixson-Crowell	Korsmeyer-Peppas	
***MSN@DOX@PDA_Cyst*** **3:1**	***R*** **^2^**					***n***
pH 4.5	0.8999	0.9136	**0.9673**	0.9092	0.9401	0.3774
pH 4.5 + 10 mM H_2_O_2_	0.8524	0.8893	**0.9724**	0.8775	0.9229	0.3917
pH 4.5 + 10 mM GSH	0.8444	0.8976	**0.9664**	0.8811	0.9436	0.2988
pH 5.5	0.8582	0.8788	**0.9748**	0.8721	0.9428	0.4422
pH 5.5 + 10 mM H_2_O_2_	0.9317	0.961	**0.9808**	0.9526	0.9478	0.3115
pH 5.5 + 10 mM GSH	0.8889	0.9327	**0.9649**	0.92	0.9048	0.448
pH 7.5	0.8239	0.8521	**0.9716**	0.843	0.9142	0.4306
pH 7.5 + 10 mM H_2_O_2_	0.8025	0.8551	**0.9603**	0.8382	0.8822	0.3588
pH 7.5 + 10 mM GSH	0.8844	0.9409	**0.9792**	0.9248	0.9281	0.4082
***MSN@DOX@PDA_Cyst*** **1:3**	***R*** **^2^**					***n***
pH 4.5	0.8332	0.8451	**0.9775**	0.8412	0.8947	0.5236
pH 4.5 + 10 mM H_2_O_2_	0.9385	0.9529	**0.9777**	0.9484	0.9591	0.5052
pH 4.5 + 10 mM GSH	0.8225	0.8636	**0.9606**	0.8504	0.9074	0.3058
pH 5.5	0.8271	0.8491	**0.9659**	0.8419	0.9186	0.3401
pH 5.5 + 10 mM H_2_O_2_	0.8948	0.9236	**0.9832**	0.9146	0.9479	0.4015
pH 5.5 + 10 mM GSH	0.8293	0.874	**0.9688**	0.8597	0.9197	0.3824
pH 7.5	0.8336	0.8602	**0.9787**	0.8516	0.9399	0.3408
pH 7.5 + 10 mM H_2_O_2_	0.7828	0.8412	**0.9443**	0.8226	0.8676	0.396
pH 7.5 + 10 mM GSH	0.8413	0.9147	**0.9754**	0.8925	0.9302	0.3868

**Table 3:** Kinetic models used to describe the release of Sorafenib (SO) from materials obtained.

Receptor Medium	Zero-Order	First-Order	Higuchi	Hixson-Crowell	Korsmeyer-Peppas	
***MSN@SO@PDA_Cyst*** **3:1**	***R*** **^2^**					***n***
pH 4.5	0.5905	0.6029	**0.8207**	0.5988	0.7747	0.2639
pH 4.5 + 10 mM H_2_O_2_	0.8641	0.904	**0.9644**	0.8915	0.9335	0.3832
pH 4.5 + 10 mM GSH	0.8455	0.9206	**0.9692**	0.8982	0.9334	0.3813
pH 5.5	0.7551	0.7788	**0.9188**	0.771	0.8713	0.3132
pH 5.5 + 10 mM H_2_O_2_	0.8025	0.8466	**0.9513**	0.8324	0.9194	0.3404
pH 5.5 + 10 mM GSH	0.813	0.8818	**0.9641**	0.8604	0.8895	0.3717
pH 7.5	0.7602	0.7755	**0.8914**	0.7705	0.8929	0.2319
pH 7.5 + 10 mM H_2_O_2_	0.9389	0.9513	**0.9971**	0.9473	0.995	0.2195
pH 7.5 + 10 mM GSH	0.7032	0.806	**0.8914**	0.7732	0.7876	0.3689
***MSN@SO@PDA_Cyst*** **1:3**	***R^2^***					***n***
pH 4.5	0.9098	0.9275	**0.9649**	0.9219	0.9088	0.4424
pH 4.5 + 10 mM H_2_O_2_	0.8976	0.9419	**0.9777**	0.9287	0.9534	0.3584
pH 4.5 + 10 mM GSH	0.9121	0.9632	**0.9745**	0.9514	0.9602	0.3579
pH 5.5	0.9411	0.9596	**0.9832**	0.9539	0.9837	0.3117
pH 5.5 + 10 mM H_2_O_2_	0.8105	0.8642	**0.9707**	0.8471	0.9382	0.3114
pH 5.5 + 10 mM GSH	0.8283	0.9277	**0.9677**	0.8995	0.9187	0.3654
pH 7.5	0.7786	0.7997	**0.8977**	0.7928	0.8497	0.2551
pH 7.5 + 10 mM H_2_O_2_	0.7999	0.8395	**0.9591**	0.8267	0.9455	0.2708
pH 7.5 + 10 mM GSH	0.8823	0.9502	**0.9717**	0.9316	0.9472	0.4206

## Data Availability

Data available upon request.

## Supplementary Materials

The following supporting information can be downloaded at: https://www.mdpi.com/article/10.3390/molecules31142413/s1, Figure S1. SEM images of polymer: PDA_Cyst 100:1 (A); PDA_Cyst 10:1 (B); PDA_Cyst 5:1 (C); PDA_Cyst 1:100 (D); PDA_Cyst 1:10 (E) and PDA_Cyst 1:5 (F); Figure S2. The XPS spectra of C, S, N and O recorded for copolymer: PDA_Cyst 1:3; Figure S3. Zeta potential values of materials prepared with different cystamine contents; Figure S4. TGA/DTG curves for the nanoparticles: PDA_Cyst 3:1 (A); PDA_Cyst 1:3 (B); PDA_Cyst 10:1 (C); PDA_Cyst 1:10 (D); PDA_Cyst 100:1 (E); PDA_Cyst 1:100 (F); TGA (red)/DTG (blue) curve; Figure S5. SEM images of MSN nanoparticles (A) and PDA nanoparticles (B); Figure S6. FT-IR spectra of MSN nanoparticles (A) and PDA nanoparticles (B); Figure S7. TEM analysis of the MSN@PDA_Cyst 1:3 copolymer; Figure S8. EDX elemental mapping of the MSN@PDA_Cyst 1:3 copolymer; Figure S9. TEM analysis of the MSN@PDA_Cyst 3:1 copolymer; Figure S10. EDX elemental mapping of the MSN@PDA_Cyst 3:1 copolymer; Figure S11. TEM analysis of the MSN@PDA_Cyst 1:3 copolymer after 72 h incubation in pH 4.5 buffer containing GSH; Figure S12. TEM analysis of the MSN@PDA_Cyst 3:1 copolymer after 72 h incubation in pH 4.5 buffer containing GSH; Figure S13. SEM images of the MSN@PDA_Cyst 1:3 copolymer: after 72 h incubation in pH 4.5 buffer containing GSH (A); after 72 h incubation in pH 4.5 buffer containing H_2_O_2_ (B); after 72 h incubation in pH 7.5 buffer containing GSH (C); Figure S14. SEM images of the MSN@PDA_Cyst 3:1 copolymer: after 72 h incubation in pH 4.5 buffer containing GSH (A); after 72 h incubation in pH 4.5 buffer containing H_2_O_2_ (B); after 72 h incubation in pH 7.5 buffer containing GSH (C).
