# Supplementary material for: Dual-Stimuli Responsive Cystamine-Modified Polydopamine Coatings as Payload Gatekeepers
Source: Molecules. 2026 Jul 9;31(14):2413. doi: 10.3390/molecules31142413 (PMC13416288; doi:10.3390/molecules31142413)
Supplement: Supplementary file 1 [file molecules-31-02413-s001.zip › molecules-4367846-supplementary.pdf]

Supporting Information

Dual-stimuli Responsive Cystamine-Modified Polydopamine Coatings as Payload Gatekeepers

Sylwia Ostrowska,[a] Monika Szukowska,[a] Yeonho Kim,[b] Radosław Mrówczyński*[a]

[a]*Faculty of Chemistry, Adam Mickiewicz University, Uniwersytetu Poznańskiego St. 8, 61-614 Poznań, Poland*

[b] *Department of Chemistry, Gachon University, Seongnam 13120, Republic of Korea*

* *radoslaw.mrowczynski@amu.edu.pl*

Table of contents

Figure S1. SEM images of polymer: PDA_Cyst 100:1 (**A**); PDA_Cyst 10:1 (**B**); PDA_Cyst 5:1 (**C**); PDA_Cyst 1:100 (**D**); PDA_Cyst 1:10 (**E**) and PDA_Cyst 1:5 (**F**)…………………………………...S3
Figure S2. The XPS spectra of C, S, N and O recorded for copolymer: PDA_Cyst 1:3……………….S4
Figure S3. Zeta potential values of materials prepared with different cystamine contents…………...S4
Figure S4. TGA/DTG curves for the nanoparticles: PDA_Cyst 3:1 (**A**); PDA_Cyst 1:3 (**B**); PDA_Cyst 10:1 (**C**); PDA_Cyst 1:10 (**D**); PDA_Cyst 100:1 (**E**); PDA_Cyst 1:100 (**F**); TGA (red)/DTG (blue) curve ………………………………………………………………………………………...S5
Figure S5. SEM images of MSN nanoparticles (**A**) and PDA nanoparticles (**B**)………………………S5
Figure S6. FT-IR spectra MSN nanoparticles (**A**) and PDA nanoparticles (**B**)………………………..S6
Figure S7. TEM analysis of the MSN@PDA_Cyst 1:3 copolymer…………………………………....S6
Figure S8. EDX elemental mapping of the MSN@PDA_Cyst 1:3 copolymer….……………………..…S7
Figure S9. TEM analysis of the MSN@PDA_Cyst 3:1copolymer…………………………………....S7
Figure S10. EDX elemental mapping of the MSN@PDA_Cyst 3:1 copolymer………………………S8
Figure S11. TEM analysis of the MSN@PDA_Cyst 1:3 copolymer after 72 h incubation in pH 4.5 buffer containing GSH…………………………………………………………………………....S8
Figure S12. TEM analysis of the MSN@PDA_Cyst 3:1 copolymer after 72 h incubation in pH 4.5 buffer containing GSH…………………………………………………………..…………………S9
Figure S13. SEM images of the MSN@PDA_Cyst 1:3 copolymer: after 72 h incubation in pH 4.5 buffer containing GSH (**A**); after 72 h incubation in pH 4.5 buffer containing H_2O_2 (**B**); after 72 h incubation in pH 7.5 buffer containing GSH (**C**)……………………………….…………………………………..S9
Figure S14. SEM images of the MSN@PDA_Cyst 3:1 copolymer: after 72 h incubation in pH 4.5 buffer containing GSH (**A**); after 72 h incubation in pH 4.5 buffer containing H_2O_2 (**B**); after 72 h incubation in pH 7.5 buffer containing GSH (**C**)……………………………………………………….…S10

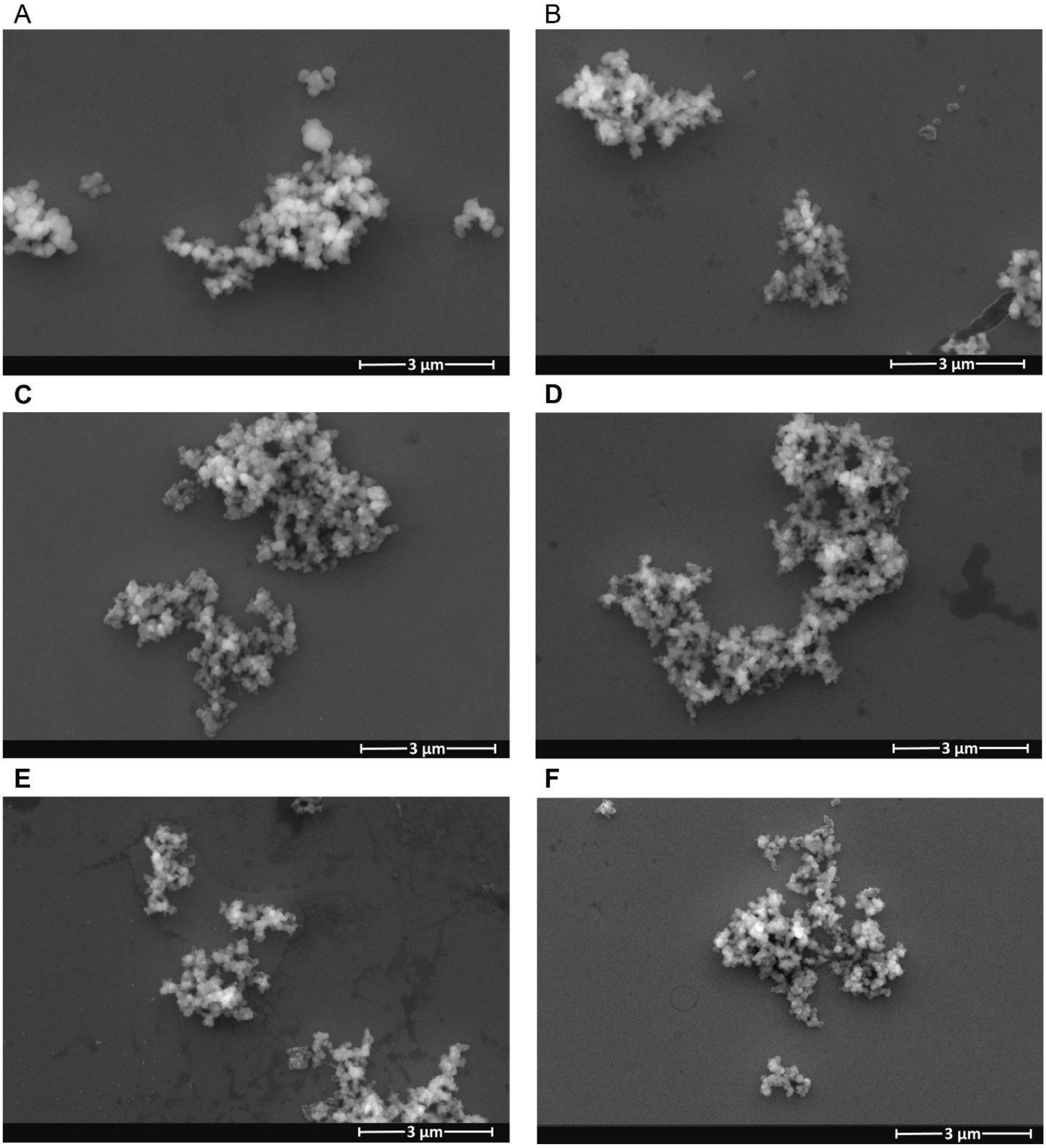

Figure S1. SEM images of copolymer: PDA_Cyst 100:1 (**A**); PDA_Cyst 10:1 (**B**); PDA_Cyst 5:1 (**C**); PDA_Cyst 1:100 (**D**); PDA_Cyst 1:10 (**E**) and PDA_Cyst 1:5 (**F**).

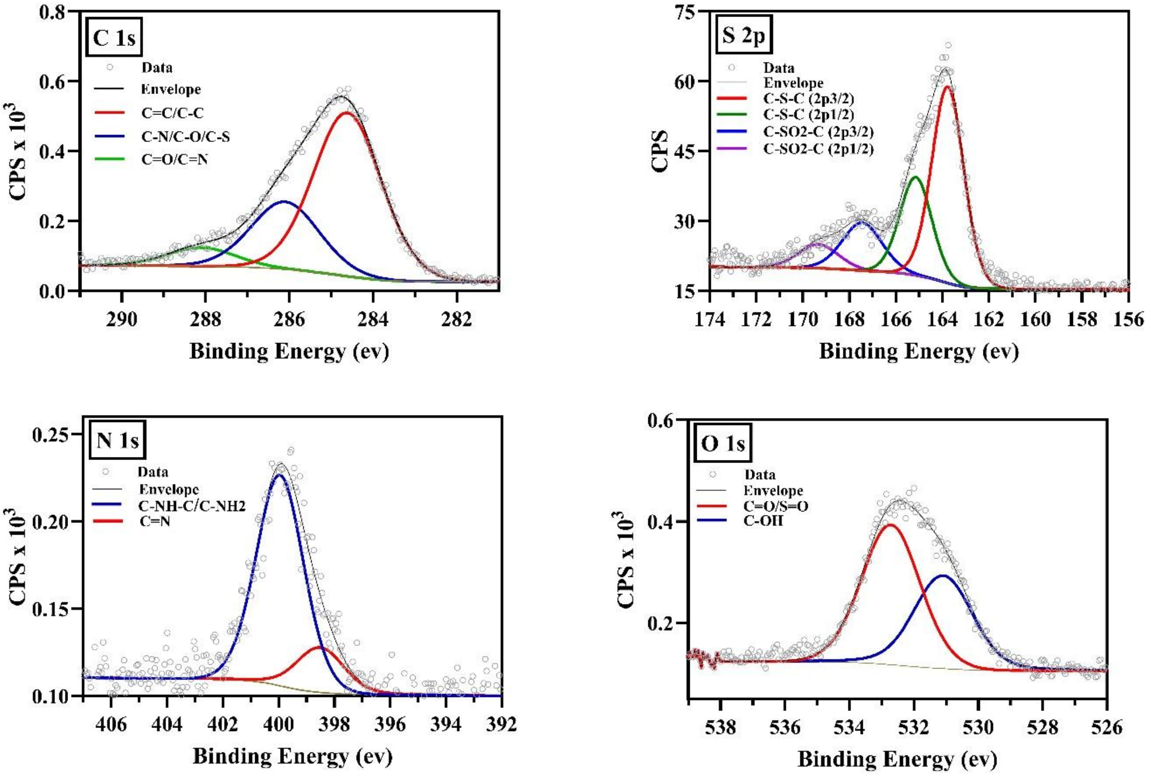

Figure. S2 The XPS spectra of C, S, N and O recorded for copolymer: PDA_Cyst 1:3.

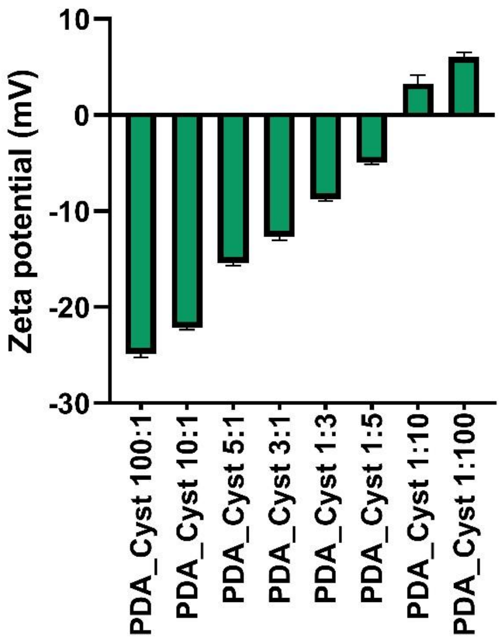

Figure S3. Zeta potential values of materials prepared with different cystamine contents.

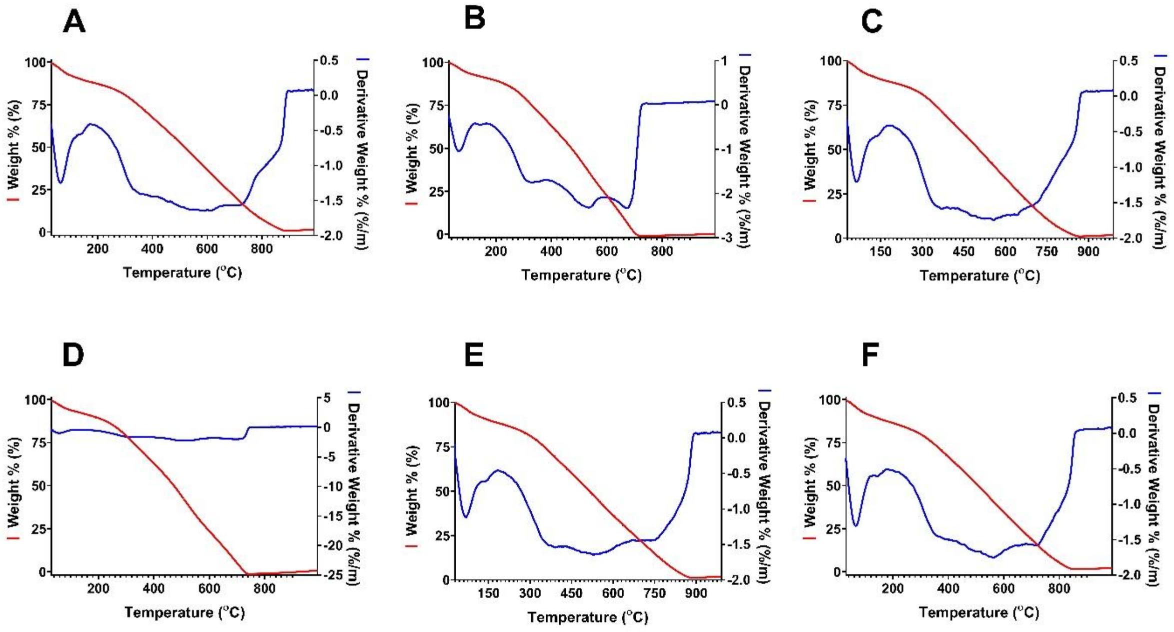

Figure S4. TGA/DTG curves for the nanoparticles: PDA_Cyst 3:1 (**A**); PDA_Cyst 1:3 (**B**); PDA_Cyst 10:1 (**C**); PDA_Cyst 1:10 (**D**); PDA_Cyst 100:1 (**E**); PDA_Cyst 1:100(**F)**; TGA (red)/ DTG (blue) curve.

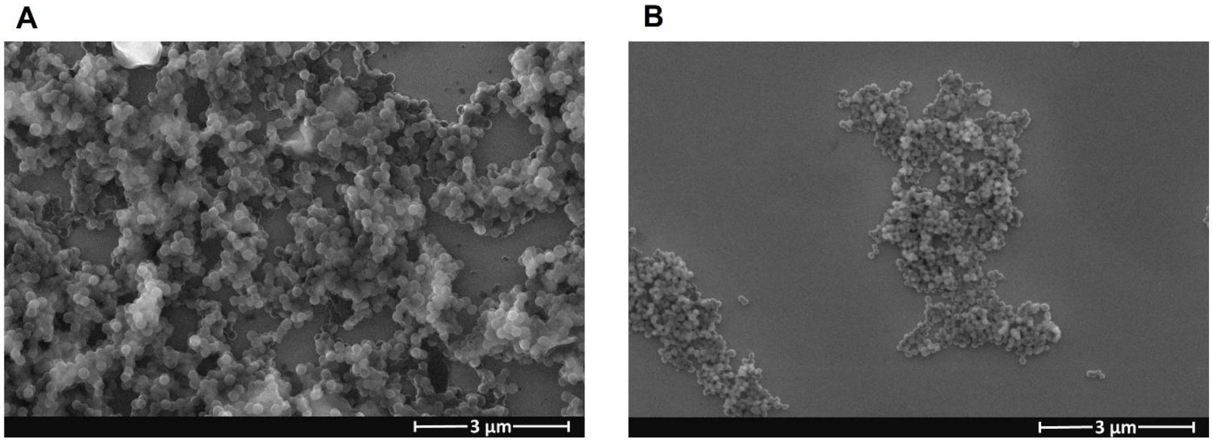

Figure S5. SEM images of MSN nanoparticles (**A**) and PDA nanoparticles (**B**).

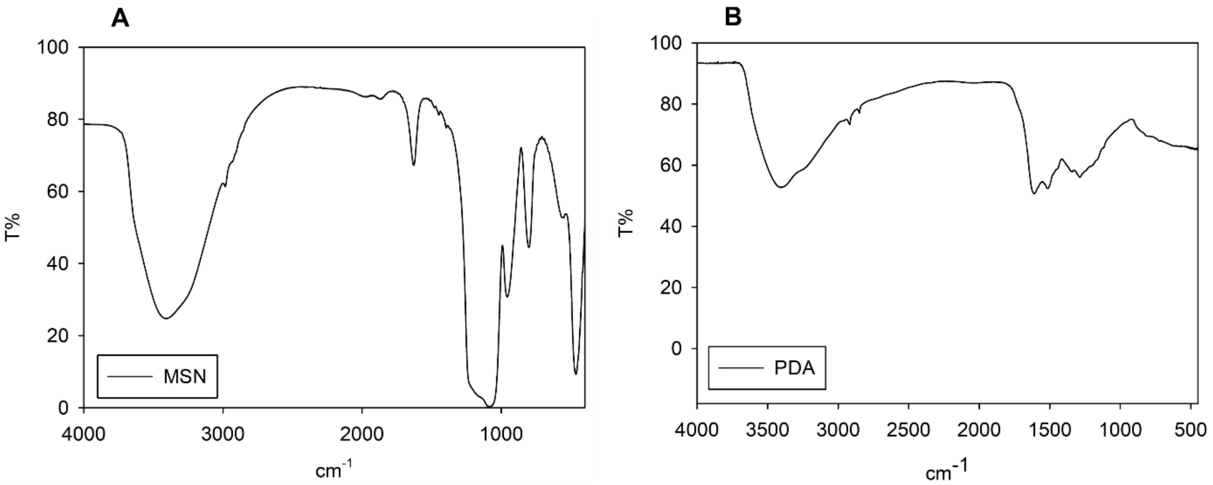

Figure S6. FT-IR spectra MSN nanoparticles (**A**) and PDA nanoparticles (**B**).

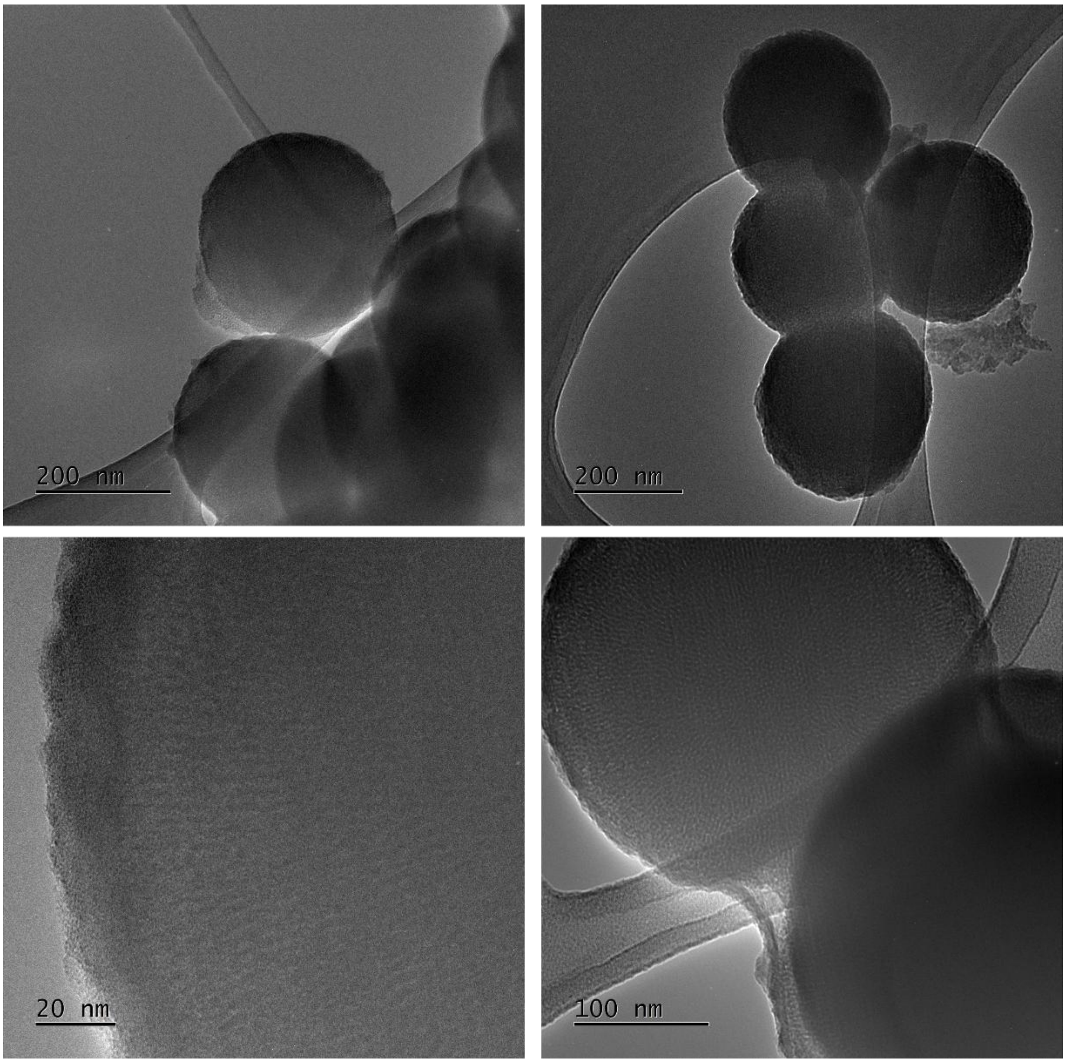

Figure S7. TEM analysis of the MSN@PDA_Cyst 1:3 copolymer.

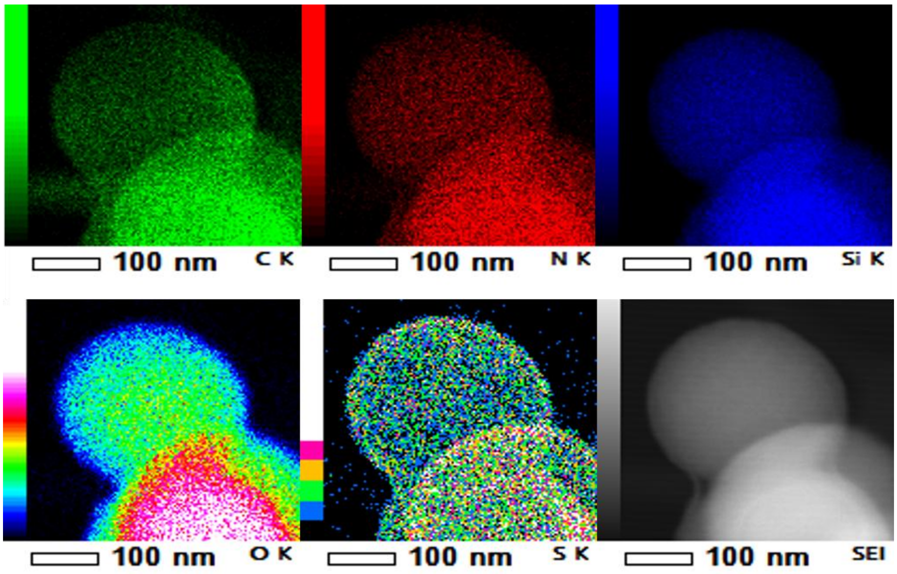

Figure S8. EDX elemental mapping of the MSN@ PDA_Cyst 1:3 copolymer.

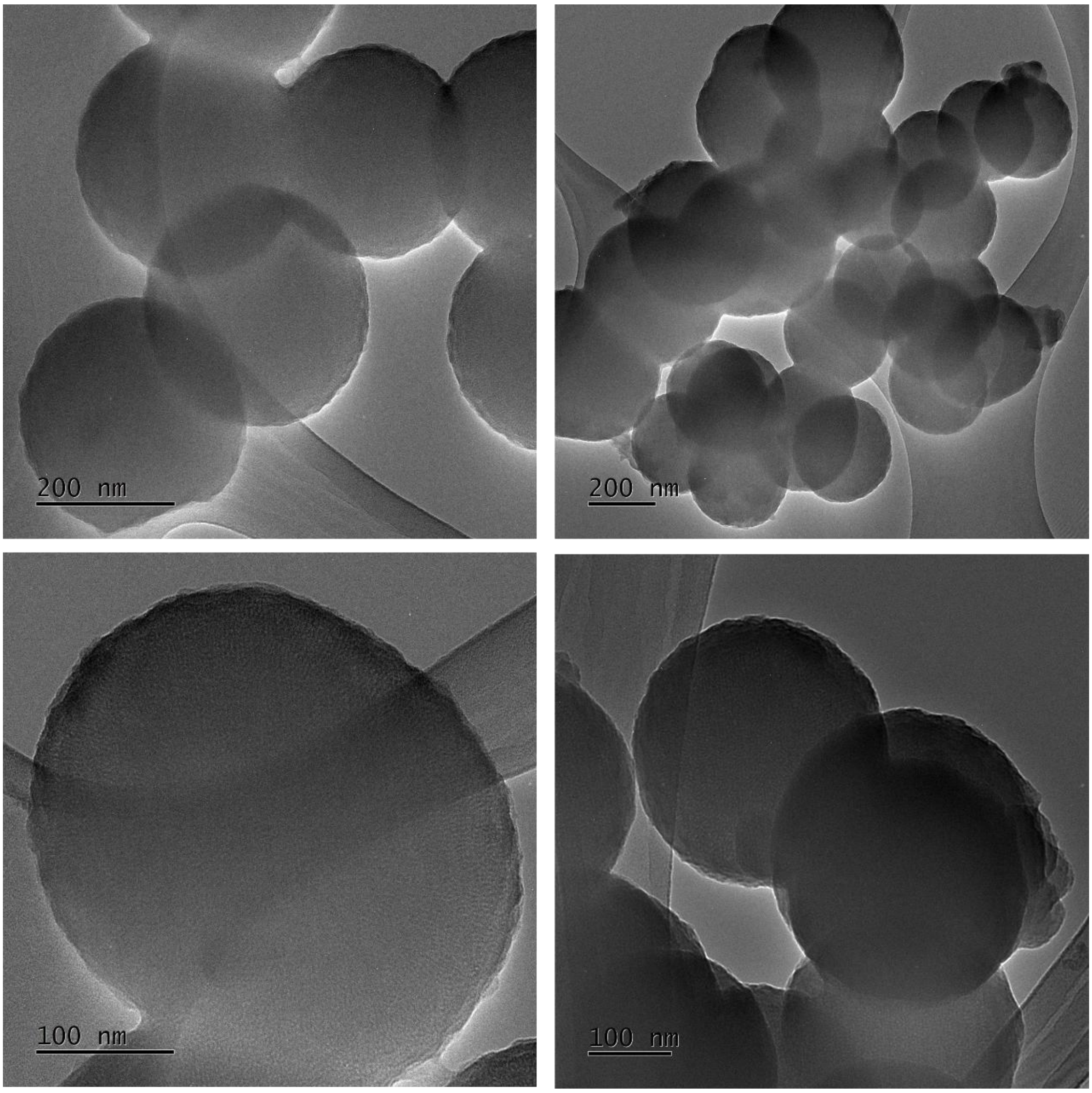

Figure S9. TEM analysis of the MSN@PDA_Cyst 3:1 copolymer.

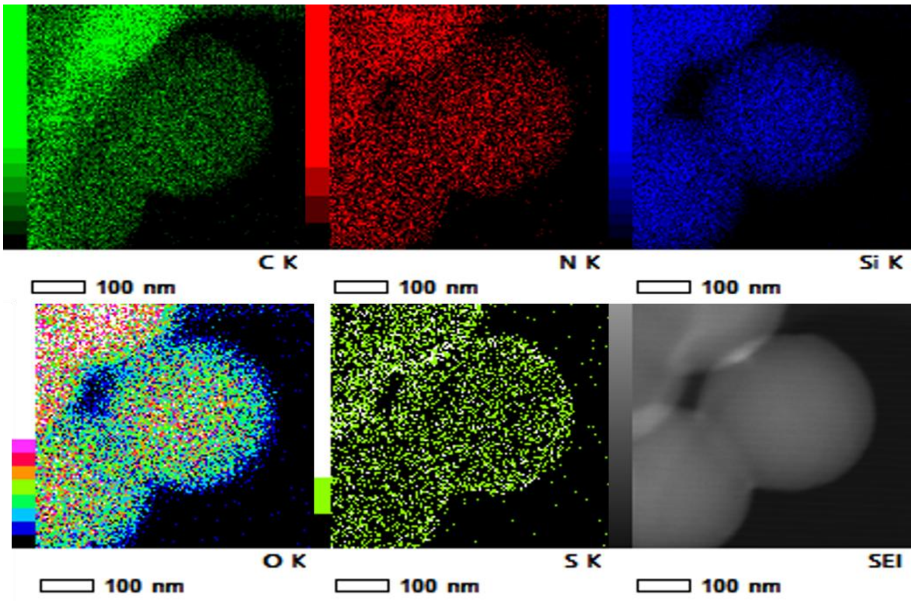

Figure S10. EDX elemental mapping of the MSN@PDA_Cyst 3:1 copolymer.

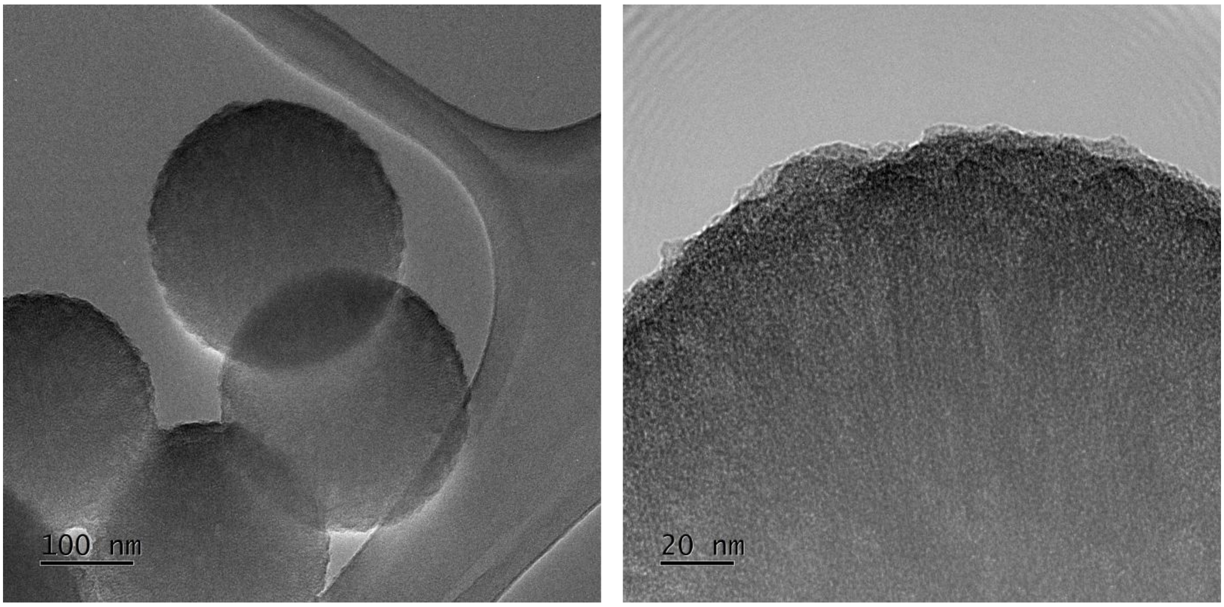

Figure S11. TEM analysis of the MSN@PDA_Cyst 1:3 copolymer after 72 h incubation in pH 4.5 buffer containing GSH.

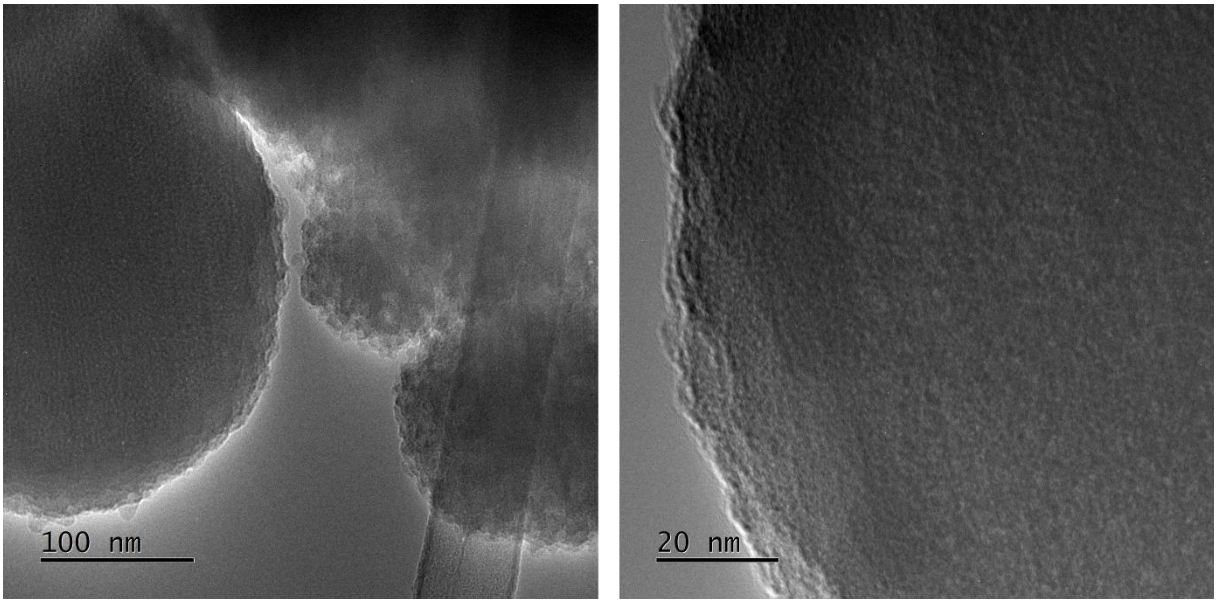

Figure S12. TEM analysis of the MSN@PDA_Cyst 3:1 copolymer after 72 h incubation in pH 4.5 buffer containing GSH.

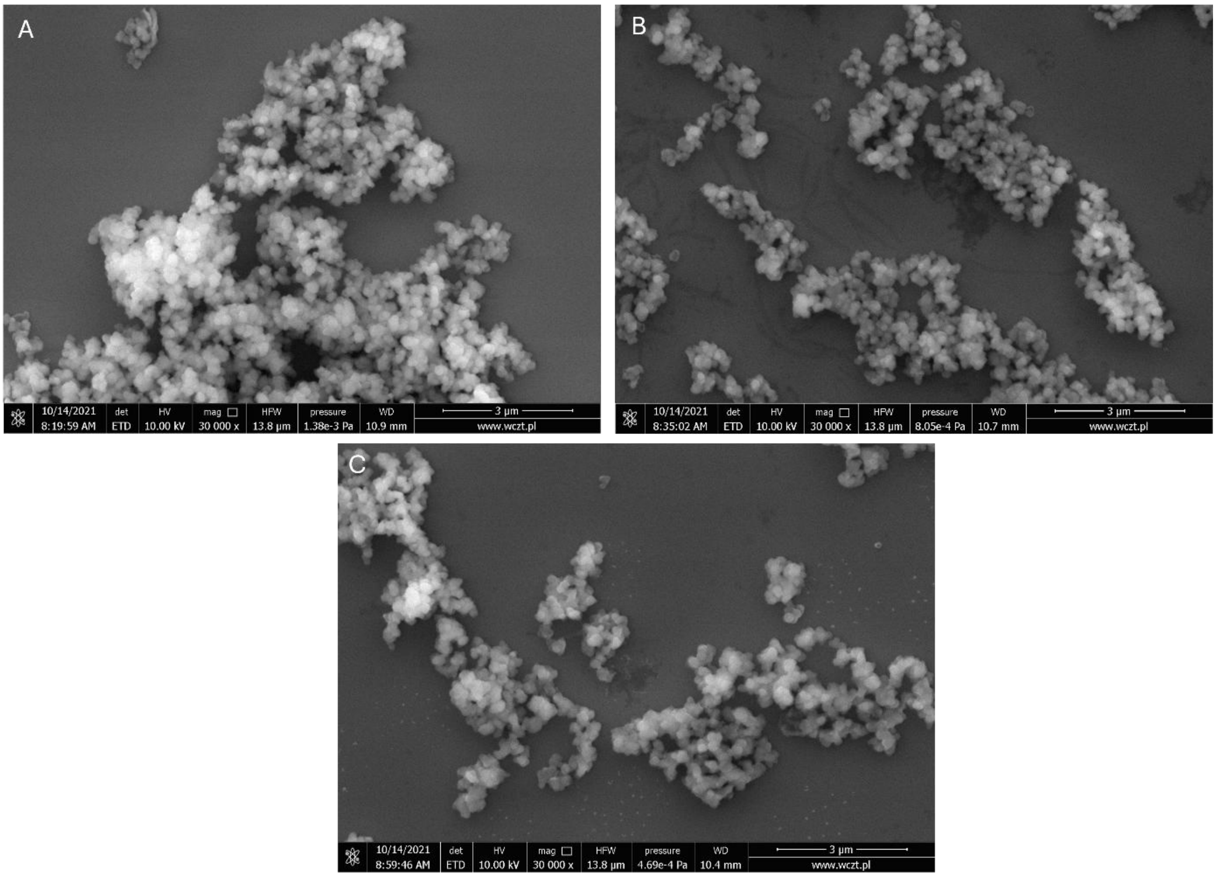

Figure S13. SEM images of the PDA_Cyst 1:3 copolymer: after 72 h incubation in pH 4.5 buffer containing GSH (**A**); after 72 h incubation in pH 4.5 buffer containing H_2O_2 (**B**); after 72 h incubation in pH 7.5 buffer containing GSH (**C**).

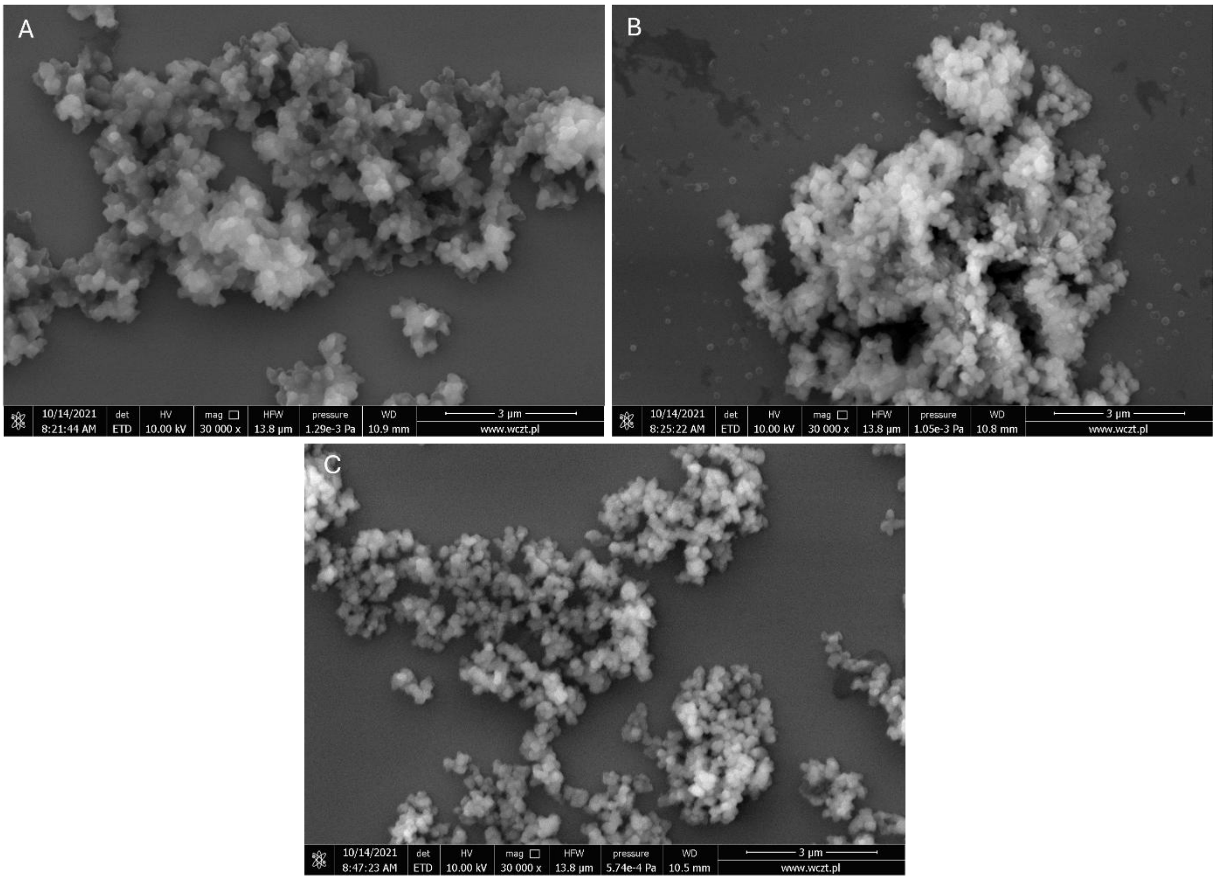

Figure S14. SEM images of the PDA_Cyst 3:1 copolymer: after 72 h incubation in pH 4.5 buffer containing GSH (**A**); after 72 h incubation in pH 4.5 buffer containing H_2O_2 (**B**); after 72 h incubation in pH 7.5 buffer containing GSH (**C**).
